# Evidence of increasing wildfire damage with decreasing property price in Southern California fires

**DOI:** 10.1371/journal.pone.0300346

**Published:** 2024-04-24

**Authors:** Erin Conlisk, Van Butsic, Alexandra D. Syphard, Sam Evans, Megan Jennings

**Affiliations:** 1 Research was Performed while at Point Blue Conservation Science, Petaluma, California, United States of America; 2 Conservation Biology Institute, Corvallis, OR, United States of America; 3 UC Berkeley, Environmental Science, Policy, and Management, Berkeley, California, United States of America; 4 Mills College at Northeastern University, Oakland, California, United States of America; 5 San Diego State University, San Diego, California, United States of America; University of Maryland at College Park, UNITED STATES

## Abstract

Across the Western United States, human development into the wildland urban interface (WUI) is contributing to increasing wildfire damage. Given that natural disasters often cause greater harm within socio-economically vulnerable groups, research is needed to explore the potential for disproportionate impacts associated with wildfire. Using Zillow Transaction and Assessment Database (ZTRAX), hereafter “Zillow”, real estate data, we explored whether lower-priced structures were more likely to be damaged during the most destructive, recent wildfires in Southern California. Within fire perimeters occurring from 2000–2019, we matched property price data to burned and unburned structures. To be included in the final dataset, fire perimeters had to surround at least 25 burned and 25 unburned structures and have been sold at most seven years before the fire; five fires fit these criteria. We found evidence to support our hypothesis that lower-priced properties were more likely to be damaged, however, the likelihood of damage and the influence of property value significantly varied across individual fire perimeters. When considering fires individually, properties within two 2003 fires–the Cedar and Grand Prix-Old Fires–had statistically significantly decreasing burn damage with increasing property value. Occurring in 2007 and later, the other three fires (Witch-Poomacha, Thomas, and Woolsey) showed no significant relationship between price and damage. Consistent with other studies, topographic position, slope, elevation, and vegetation were also significantly associated with the likelihood of a structure being damaged during the wildfire. Driving time to the nearest fire station and previously identified fire hazard were also significant. Our results suggest that further studies on the extent and reason for disproportionate impacts of wildfire are needed. In the meantime, decision makers should consider allocating wildfire risk mitigation resources–such as fire-fighting and wildfire structural preparedness resources–to more socioeconomically vulnerable neighborhoods.

## Introduction

Wildfire is a key mechanism of ecosystem structure and function, and changes in wildfire regimes can pose a significant threat to wildland resources and human communities. Human development in the wildland urban interface (WUI)–areas where residential development is adjacent to or mixed within natural lands–has led to increased frequency and shifting spatial patterns of human-ignited wildfire [1, 2]. In turn, these fires have become increasingly destructive due to the arrangement and location of homes within the WUI [3]. Currently it is estimated that almost 50 million homes are located within the WUI in the United States, with 1 million more being built every 3 years [4]. In California, the overall economic impact of wildfires in 2018 was estimated to be 1.5% of the state’s GDP [5]. Mitigating wildfire structure loss is particularly complex in Southern California, where a population of 24 million people is adjacent to two very different ecosystems–shrublands and montane forests–experiencing divergent changes from historical fire regimes (e.g. increasing and decreasing fire frequency, respectively) [6]. Nevertheless, the majority of structure losses in the state occur within non-forested vegetation types, particularly chaparral shrublands [7].

The socio-economic impact of natural disasters, such as wildfires, depends on the biophysical properties of the hazard as well as the ability of the human community to respond [8]. Overall, natural disasters tend to have a bigger impact on less affluent communities [9]. Acknowledging the potential for disproportionate social impacts of natural and climate change-induced disasters, a number of recent studies have investigated the relationships between environmental justice and wildfire, although the literature remains relatively scarce [10]. Studies typically focus on adaptive management, wildfire insurance, risk mitigation, air pollution, and community perceptions of the wildland urban interface. To our knowledge, no studies have explored whether lower-priced properties are more likely to be damaged during a wildfire.

While still a relatively young research topic, studies suggest that more socio-economically vulnerable communities could also be more vulnerable to the impacts of wildfire. Using U.S. Forest Service wildfire hazard and census tract data, a recent study found that wildfire vulnerability across the U.S. is spread unequally across different demographic groups, with majority Black, Hispanic or Native American census tracts experiencing approximately 50% greater wildfire vulnerability compared to White and Asian census tracts [11, 12]. In California, census tracts associated with lower home price occurred in locations that burned more frequently [13, 14]. Similarly, locations with greater Native American populations were also exposed to more wildfire [13]. Wigtil et al. [15] mapped locations in the coterminous U.S. where high wildfire hazard overlapped with social vulnerability. While most locations in Southern California were mapped as having high fire hazard, these hazardous locations were split evenly among low and moderate social vulnerability [15]. However, this map was at low resolution and could miss pockets of high social vulnerability within larger areas of low and moderate vulnerability. Studies with high resolution vulnerability data are needed.

Compared to their more affluent neighbors, socio-economically vulnerable populations may have different reasons for living in the WUI and lower wildfire preparedness. Many homeowners move into the wildland urban interface because of a desire to be closer to natural amenities [11, 15]. In contrast, some homeowners move to the wildland urban interface because they are priced-out of urban centers, a phenomenon that accelerated during the COVID19 pandemic [16]. Land-use restrictions and zoning may also contribute to migration into WUI and fire-prone locations [17]. Still others live in the WUI because they were involved in current or past natural resource extraction (e.g. ranching, logging, and mining; [18]). These different reasons for residing in the WUI create different priorities for wildfire home preparedness, often called “fire hardening,” which includes activities to prevent the ignition of flying embers (e.g. screening or enclosing rain gutters, vents, and eaves to prevent flammable vegetation debris from accumulating). Homeowners who moved to the WUI for the natural amenities may not want to create defensible space–defined as landscape management around a structure to reduce flammability and flame lengths–because it will make the landscape look less “natural.” In contrast, homeowners who have been priced-out of urban centers or are part of a declining resource-extraction industry, may find fire hardening expenses prohibitively costly, where lack of financial resources is an often cited barrier to fire hardening [18, 19]. While these different WUI demographics have been described in a number of studies [18–22], more research is needed to determine whether choices about home hardening lead to disproportionate impacts of wildfire across different socio-economic groups.

Several environmental conditions and landscape characteristics have been found to increase the likelihood of structure loss during wildfire. Looking at structure loss across the U.S. and in case studies in California and Colorado, Alexandre et al. [23, 24] found that variables related to topography and building arrangement were more influential than vegetation-related variables, and that vegetation connectivity was more important than vegetation type. Other studies showed that housing arrangement and location were the most important variables associated with structure loss; however, the relative importance of different variables varies by region and scale [25–28]. At local spatial scales, the creation of defensible space can significantly increase structure survival during wildfires; but the significant effect was not observed beyond 30m (100ft) [29]. Building materials and structural characteristics can also significantly improve the potential for structure survival during wildfire [30–33].

Because of the increasing risk of wildfire and increasing wealth inequality, understanding the relationship between social vulnerability and structure damage is important for wildfire management and planning. For example, information about if, where, and how wildfire results in disproportionate socio-economic impacts can help target mitigation resources and activities, particularly by informing prioritization of wildfire resilience resources. As a first step, we use property price as an indicator of socio-economic conditions and explore whether wildfire structure damage is related to property price within a wildfire perimeter. Specifically, we associated remotely sensed data on structure damage due to wildfire with Zillow Transaction and Assessment Database (ZTRAX), hereafter “Zillow”. We hypothesized that lower priced properties were more likely to be damaged in a wildfire compared to higher priced properties and that environmental variables would influence structure damage, consistent with prior studies [23–27, 29, 34]. This analysis will provide one of the first studies of the influence of property value on the likelihood of structure damage during wildfire.

## Methods

### Study area

Our target area was the South Coast ecoregion of Southern California, a geographically and biologically diverse region with a Mediterranean climate and a large WUI footprint [35]. A broad set of habitats, including grasslands, coastal sage and chaparral shrublands, oak woodlands, and conifer forests contribute to a variation in fire behavioral characteristics within the area. The study area included portions of San Bernardino, Santa Barbara, Ventura, Los Angeles, Orange, Riverside, and San Diego Counties. Across Southern California, most wildfires are human-ignited [36]. Two types of fires are prominent in Southern California, each contributing roughly equally to area burned [37]. Fast-spreading, Santa Ana wind-driven fires typically occur within shrublands in October through April and cause the bulk of the economic costs due to property damage. Non-Santa Ana wind-driven fires move more slowly, are typically sensitive to age-dependent forest fuels, cause much less property damage, and are responsible for 70% of suppression costs [37].

### Modeling approach and property prices

**Fig 1:**
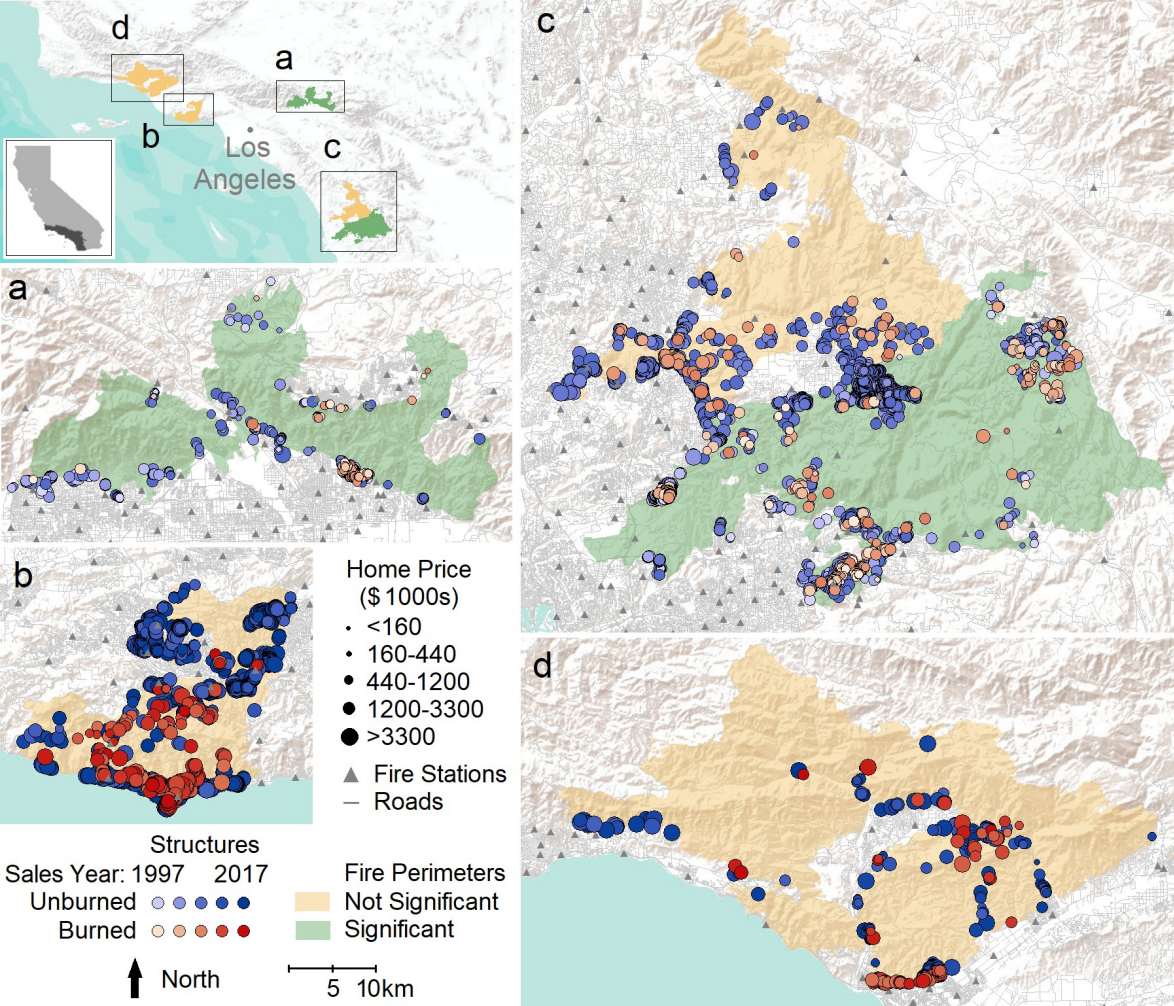
Map of study area. Unburned (shades of blue) and burned (shades of red) structures are shown on top of fire perimeters (yellow and green). Fires are as follows: (a) 2003 Grand Prix-Old Complex, (b) 2018 Woolsey, (c) 2007 Witch-Poomacha (yellow) and 2003 Cedar (green), and (d) 2017 Thomas. Points are sized according to the property sales price (larger points mean greater sales price–on a natural log scale). Point color intensity reflects when the property was sold within the period 1997–2017. Fires colored in yellow did not show a significant relationship between property price and likelihood of burn damage. Fires colored in green had a significant relationship of decreasing burn damage with greater property price. Fire stations (gray triangles) and roads (gray lines) are also shown, along with an inset of the study region’s location within California. All maps (a)-(d) are on the same spatial scale. Topographic base map provided by [39].

We explored whether property price was a significant predictor of wildfire structure damage in the South Coast ecoregion of California (Fig 1 inset). To address this question, we assigned property prices (from Zillow) to burned and unburned structures controlling for landscape characteristics that influence wildfire risk. We attempted to follow the prescribed best practices for use of Zillow data according to Nolte et al. (In Press) [38].

Structures were assigned to the “burned” category through remotely sensed image analysis. Typically, if burn damage was detectable in images, the damage was substantial, resulting in complete structure destruction and loss. However, properties can have multiple structures on a parcel and we cannot be sure that remotely sensed destroyed structures were a property’s primary residence (as opposed to structures such as detached garages, barns, or sheds). Thus, while we assume burned structures were completely destroyed, we still refer to burn “damage” and not “destruction” or “loss.”

We obtained data on burned and unburned structures within fire perimeters occurring from 2000–2010 from the appendix of [23]. After 2010, spatial data from remotely sensed unburned structures came from Microsoft [40] and burned structures came from digital mapping of burned structures (similar to the methods of [23] and) described in [26]. Next we associated these structural observations with parcel boundaries by overlaying structural coordinates with parcel boundaries and removing any duplicate parcels (because there can be more than one structure on a property). S1 Table in S1 File shows the availability of remotely sensed structure data.

To associate the structure data with the property price data, we matched the structure’s parcel number with the parcel numbers in the Zillow database. We extracted Zillow sale price (proprietary “Zestimates” were not available), year of sale, year built, and property use, where available. As we sought to examine burn damage probability on residential homes, we removed non-residential property uses such as condominiums, commercial, agricultural, and unimproved lots. While our intent is to describe the damage of owners’ homes, we cannot be sure that all single-family structures are owners’ homes; thus, we refer to the sale of a “property” and not a “home”. Property prices referred to the sale of all structures on the parcel.

We then determined whether relevant sales occurred within Southern California fire perimeters. Fire perimeter data came from California Department of Forestry and Fire Protection, Fire Resources and Assessment Program website (CALFIRE FRAP) website [41]. We considered fires that occurred between 2000 and 2019 because we had structure damage data for this period and because the quality of the Zillow data improves after April 1996. When two adjacent fires merged, we considered the two fires as a single fire complex; examples include the merging of the 2007 Witch and Poomacha Fires and the 2003 Grand Prix and Old Fires.

To be included in the model, a fire must have surrounded at least 25 burned and 25 unburned structures and have been sold shortly before the fire, defined as occurring no more than seven years before the fire. We did this as a compromise between wanting to have a price that provided a good estimate of the value of the property at the time of the fire–going further back in time would make prices less reliable–and wanting to have a reasonable sample size of data. During the seven-year period before a fire, only 11–15% of structures that were later burned were sold (Table 1). With similar representation in the unburned structures, the majority of burned and unburned properties within a fire perimeter could not be included in the analysis. Overall, these filtering criteria led to a total of five fires in the final dataset: the 2003 Cedar Fire, 2003 Grand Prix-Old Complex, 2007 Witch-Poomacha Complex, 2017 Thomas Fire, and 2018 Woolsey Fire. For each of the five fire perimeters we examined, there were anywhere from 4.1 to 12.6 times as many unburned structures in the data as burned structures, averaging 8.2 times as many unburned as burned structures (Table 1).

**Table 1:** Summary statistics for the individual fires.

Fire Name	Year of Fire	Number of Unburned Structures with Sales Data	Number of Burned Structures with Sales Data	Total Number of Burned Structures^1^	Fraction with Sales Data that were Burned	Fraction of Burned Structures with Sales Data
Cedar	2003	2713	333	4847	0.109	0.069
Grand Prix-Old	2003	571	130	1139	0.185	0.114
Witch-Poomacha	2007	647	56	1867	0.080	0.030^2^
Thomas	2017	373	121	1063	0.245	0.114
Woolsey	2018	1654	190	1643	0.103	0.116

^1^ Data were obtained from CALFIRE incident reports [42]. Total number of structures are reported regardless of whether there are multiple structures on a property, likely overestimating the availability of data.

^2^ Fewer structures had sales data for the Cedar Fire because sales data from 2004–2007 were removed due to the real estate bubble.

We also removed any properties with “Year Built” occurring after a sale but before a fire, which likely indicate large remodels. Removing these observations mitigates the potential for large property remodels to alter the property’s value between the time of sale and the fire. There were also some properties that had year built after the fire; most of these values reflect post-fire reconstruction. We excluded these data in models that analyzed year built, resulting in the loss of more than 50% of data from burned structures. Because of the loss of such a large fraction of burned property data, we place the results of this model in Supplemental Information S1. In the models that did not analyze year built (which are presented in the main paper), we could still include properties with year built after the fire because the sales associated with these properties occurred before the fire.

**Fig 2:**
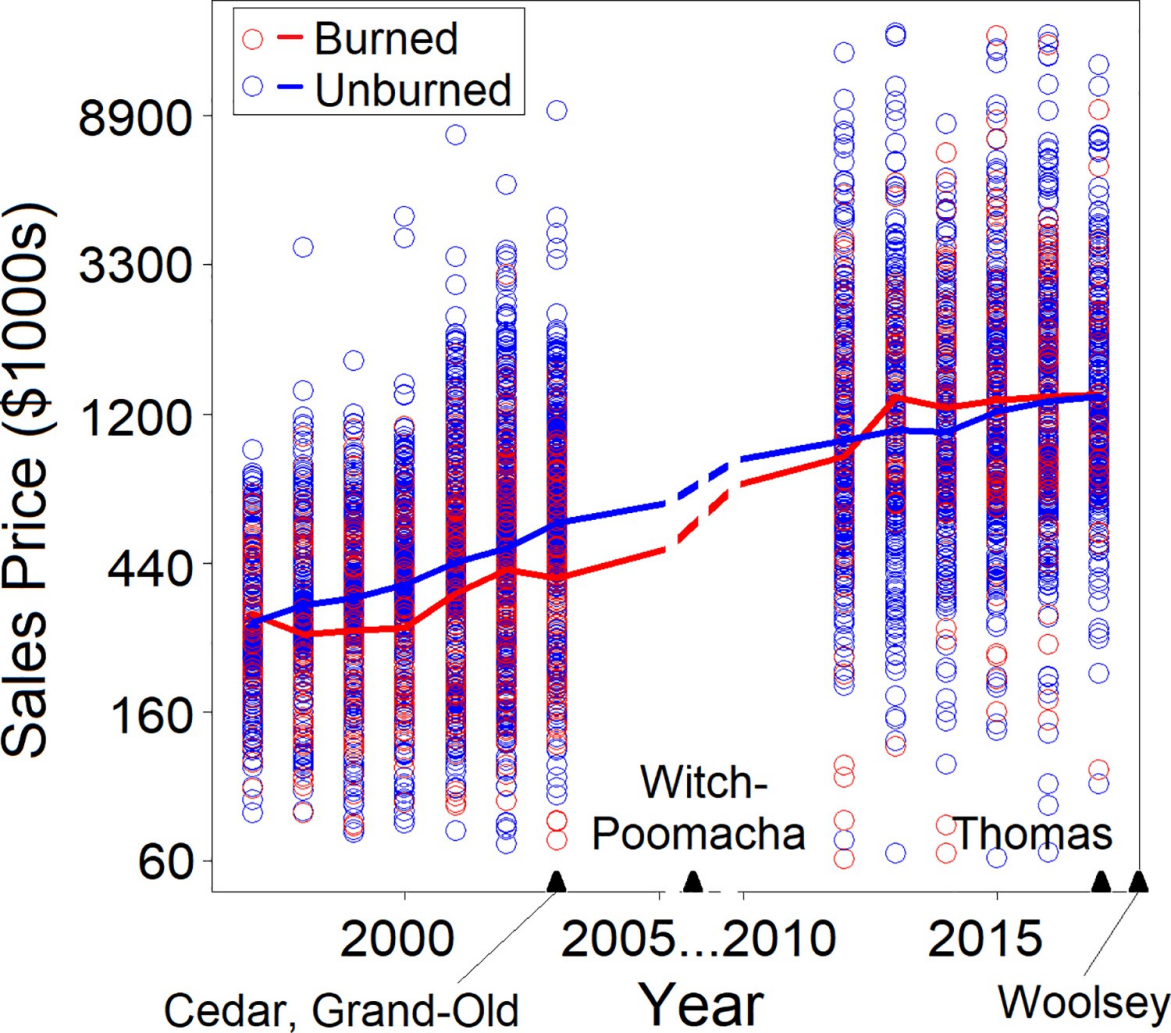
Sales price for burned and unburned structures across years. Individual property sales prices (in thousands of dollars on a log scale) are shown across years in which the properties were sold for the five fires in the dataset. The lines go through the value for the average property price for burned and unburned structures in each year. Triangles at the bottom of the figure show when each of the fires happened. Property price has been converted to 2017 dollars using the consumer price index. There is no data for 2004–2012 because these sales were removed due to the real estate bubble, recession, and post-recession recovery. Only sales that occurred within seven years prior to the fire were included.

We converted the sale price to 2017 dollars using the consumer price index for all urban consumers (CPIAUCSL) which adjusts for the annual change in the price of goods. We also included sales-year in the model to account for real estate appreciation in addition to annual changes in the price of goods. Fig 2 shows property prices across years for burned and unburned structures. To remove price outliers that may represent unusual or misclassified property uses, we only included properties with sales prices between $50,000 and $15,000,000. We also eliminated sales within the period 2004–2012 because of the potential impact of the real estate bubble, recession, and post-recession recovery. Note that we did not eliminate fires occurring between 2004–2012, provided enough sales data were available outside of this period. We chose to eliminate sales within this window because we saw clear evidence of differential impacts of the real estate bubble and recovery as a function of neighborhood home price. Lower-priced neighborhoods saw more rapid appreciation before the recession (2004–2007), a sharper decline during the recession (2007–2009), and much slower recovery after the recession (2010–2012). By 2012, the differences in the effects of the recession across neighborhoods were no longer evident in the data.

To evaluate whether our results were robust to the data filtering described above, we conducted an additional suite of analyses where we relaxed the rigor of our data-inclusion criteria, expanding our analysis to 12 of a possible 22 fires within the period 2000–2019 that had more than 100 structures destroyed (S1 Table in S1 File and S1 Fig in S1 File). Specifically, we considered (i) reducing the number of burned structures within a fire perimeter from 25 to 15, (ii) including sales that occurred any time after April 1996 but before the fire (instead of including only property sales that occurred within the seven years preceding a fire), and (iii) including data within the real estate bubble and recession period of 2004–2012. We also considered the relationship between a property’s assessed value and the probability of burn damage. The assessed value of a property is not the ideal metric of value because California Proposition 13 limits annual property appreciation to 2% unless the property is sold, in which case the sales value is used. However, using assessed value expanded our analysis, allowing us to explore an additional three fires with greater than 100 structures destroyed for a total of 15 out of 22 possible fires being explored. Given a correlation of 0.65 between sales and assessed value, we chose to present models of assessed value in Supplemental Information S1.

Finally, for the subset of data with structure construction dates (or “year built”) available, we analyzed the importance of property price in the context of the age of the structure. We performed these analyses using the five-fire dataset presented in the main paper and for the assessed value data presented in Supplemental Information S1. The results of these analyses are also presented in Supplemental Information S1.

### Wildfire covariates

To isolate the influence of property value on structure damage, we needed to control for the increased risks associated with structures built in more fire-prone locations. We selected model covariates based on previous work at national [23] and regional [25] scales that found significant associations between structure loss and the variables listed in Table 2 (and shown in S2 Fig in S1 File): topographic position index (or TPI), elevation, slope, road density, and vegetation type. While values for these variables were present in the appendix of [23], we recalculated all variables for both the data from before 2010 [23] and the additional burned and unburned structure data for later years. Specifically, at the coordinates of a structure, we extracted values from raster based maps listed in Table 2 (and shown in S2 Fig in S1 File). We also included an additional variable–time to fire station–as a measure of the availability of suppression resources. Fire hazard, vegetation type, and fire identity were “factors” (categorical data) in the model and all other variables were continuous. We aggregated hazard data into two categories: low and high (see Table 2). We also aggregated vegetation types into four categories: forest (including hardwood and conifer), shrub, herbaceous, and barren (including urban, agriculture, barren, and wetlands). Supplemental Information S1 includes additional analyses with different subsets of data, including more wildfires, to explore relationships between burn damage and assessed value and year built.

**Table 2:** Landscape characteristics used to predict fire damage. Unless otherwise noted, all raster-based maps had a 90-meter resolution.

Name	Description and source	Source
Property price	Specifically the “SalesPriceAmount”	Zillow Transaction Data: https://www.zillow.com/research/ztrax/
Year of Sale	Year of sale listed on transaction document. Specifically, we used “DocumentDate”, where available, followed by “RecordingDate”.	Zillow Transaction Data: https://www.zillow.com/research/ztrax/
Time to Fire Station	Locations of all fire stations in California. Latitude and longitude for the two closest stations were extracted and the shortest driving time was recorded–driving times were requested from Google Distance API (through the R package with eponymous function gmapsdistance).	U.S. Geological Survey, 2020 [43], Google Distance API
Elevation	In meters from the National Elevation Dataset.	U.S. Geological Survey 2009 [44]: http://viewer.nationalmap.gov/viewer/
Topographic Positon Index (TPI) at 500m	Total curvature derived from National Elevation Dataset (row above) with DEM Surface Tools (Jenness 2013).	U.S. Geological Survey 2009
Slope within 500m	Derived from National Elevation Dataset with DEM Surface Tools. Pixel values were averages across a 250m radius.	U.S. Geological Survey 2009
Road Density	The density of roads (km/km^2^) within a 500m radius from the structure.	2019 TIGER/Line Shapefiles U.S. Census Bureau
Fire Hazard	Translated into two categories: High (hazard values 4 and 5) and Low (values 3 or lower). Map was at a 30-meter resolution.	California Department of Forestry and Fire Protection (CALFIRE), Fire Resources and Assessment Program (FRAP) 2019: https://frap.fire.ca.gov/mapping/gis-data/
Vegetation Type	Translated to four categories from the “LIFE_FORM” field: herbaceous, shrub, wooded (including conifer), and urban (including water, barren, and agricultural). Map was originally a shapefile that was converted to a 90-meter raster.	FVEG (originally from FRAP 2019): https://frap.fire.ca.gov/mapping/gis-data/
Fire identity	The name of the fire within whose perimeter structures were considered.	FRAP 2019: https://frap.fire.ca.gov/mapping/gis-data/

### Statistical analysis

All hypothesis testing was done using a logistic regression in the R programming language [45]. We performed three analyses on: (i) the entire five-fire dataset, (ii) each fire considered separately, and (iii) bootstrapped subsamples of data on each fire considered separately. Coefficient estimates are expressed as log-odds ratios. Based on the model that included all fires together (or (i)), we found a significant fire by property price interaction, thus leading to the analysis of each fire separately (ii). Because there were many more unburned structures compared to burned structures, we ran 1000 bootstrapped subsamples of data (or (iii)), including 90% of burned structure data, and the same number of burned and unburned structures. With these subsamples, we explored model coefficients on property price.

## Results

**Fig 3:**
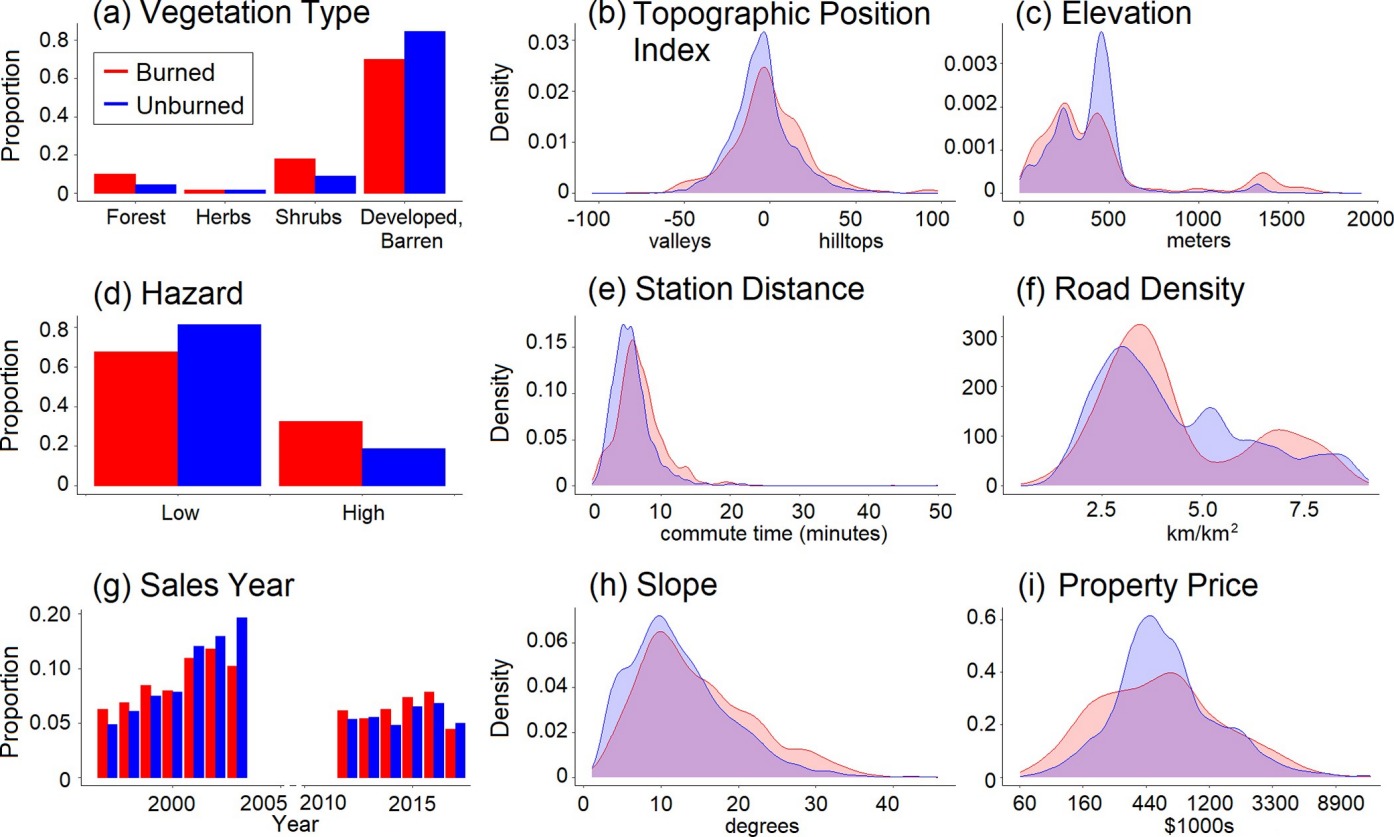
Densities of explanatory variables for burned and unburned structures. The proportion of burned and unburned structures within different land cover types (a and d), across different years (g; each red-blue bar pair representing one year of sales), and densities of covariate values for burned and unburned structures (b, c, e, f, h, and i) across the burned and unburned structures in the five fires studied.

Using the five-fire dataset, our models agreed with previous studies that found the following landscape characteristics were significant predictors of structure damage: elevation, topographic position, slope, and cover type (Table 3). Fire hazard, fire identity, and driving time between the structure and the fire station were also significant. Comparing covariate values for burned structures to unburned structures (Fig 3), a higher fraction of burned structures were in locations with high fire hazard, in forests and shrublands, on hilltops versus valleys, farther from a fire station, and with a steeper slope. In four out of five single-fire models, topographic position index, slope, and road density were significant (Table 4). In single-fire models, station distance was significant only for the Cedar Fire. Elevation and vegetation type were only significant in one and two of five of the single-fire models, respectively.

**Table 3:** Coefficient estimates and likelihood ratio test p-values for explanatory variables in the five-fire dataset. Residual deviance was 4620 on 6768 degrees of freedom, with pseudo-r^2^ = 0.084.

	Coefficient Estimate	Coefficient Std. Error	LR Chisq	Df	Pr(>Chisq)
**Intercept**	2.127	1.065			
**ln(Price)**	-0.345	0.078	20.111	1	**7.3x10** ^**-6**^
**Fire Identity**			62.575	4	**<10** ^**−6**^
Cedar	2.875	1.531			
Grand Prix-Old	10.740	2.278			
Witch-Poomacha	-2.920	2.641			
Thomas	-0.363	1.795			
Woolsey	-10.330	1.602			
**Station Distance (mins driving)**	0.086	0.011	59.532	1	**<10** ^**−6**^
**Topographic Position Index (500m)**	0.012	0.002	38.408	1	**<10** ^**−6**^
Sales Year	-0.029	0.022	1.721	1	0.190
**Slope in 500m moving window**	0.035	0.006	31.959	1	**<10** ^**−6**^
**Elevation**	-0.0005	0.0002	6.259	1	**0.012**
Road Density (km/km2)	11.570	27.841	0.173	1	0.678
**Fire Hazard—High**	0.125	0.060	4.335	1	**0.037**
**Cover Type**			28.083	3	**3.5x10** ^**-6**^
Urban, Ag, Barren, Water	-0.241	0.112			
Forest	0.553	0.137			
Herbaceous	-0.556	0.227			
Shrub	0.244	0.114			
**ln(Price) x (Fire Identity)**			65.597	4	**<10** ^**−6**^
Cedar	-0.228	0.116			
Grand Prix-Old	-0.839	0.183			
Witch-Poomacha	0.185	0.196			
Thomas	0.122	0.132			
Woolsey	0.761	0.115			

**Table 4:** Coefficient estimates and likelihood ratio test p-values for explanatory variables in the individual fire models. The *r*^*2*^s for the Cedar, Grand Prix-Old, Witch-Poomacha, Thomas, and Woolsey fires are 0.14, 0.10, 0.10, 0.13, and 0.20, respectively. TPI refers to the topographic position index within a 500m window.

	Cedar Fire 2003					Grand Prix-Old Fire 2003					Witch-Poomacha Fire 2007					Thomas Fire 2017					Woolsey Fire 2018				
	Coef. Est.	Std. Error	LR Chisq	Df	Pr (>Chisq)	Coef. Est.	Std. Error	LR Chisq	Df	Pr (>Chisq)	Coef. Est.	Std. Error	LR Chisq	Df	Pr (>Chisq)	Coef. Est.	Std. Error	LR Chisq	Df	Pr (>Chisq)	Coef.Est.	Std. Error	LR Chisq	Df	Pr (>Chisq)
Intercept	3.953	1.618				4.766	3.270				-4.220	158.800				-1.443	2.669				3.181	2.100			
ln(Price)	-0.684	0.129	27.678	1	**<10** ^**−6**^	-0.980	0.263	15.217	1	**9.6x10** ^**-5**^	-0.016	0.283	0.003	1	0.956	-0.080	0.171	0.221	1	0.639	-0.171	0.124	1.901	1	0.168
Station Distance	0.193	0.024	72.47	1	**<10** ^**−6**^	0.038	0.037	0.971	1	0.324	0.078	0.041	3.463	1	0.063	0.033	0.042	0.593	1	0.441	-0.018	0.023	0.584	1	0.445
TPI	0.013	0.003	13.78	1	**0.00021**	-0.017	0.008	4.766	1	**0.029**	0.023	0.008	7.708	1	**0.005**	0.015	0.006	7.228	1	**0.0072**	0.002	0.004	0.329	1	0.566
Sales Year	-0.020	0.034	0.349	1	0.555	0.013	0.055	0.058	1	0.810	-0.235	0.182	1.662	1	0.197	-0.062	0.070	0.769	1	0.380	-0.001	0.050	0	1	0.985
Slope	0.051	0.011	19.62	1	**9.5x10** ^**-6**^	0.071	0.020	12.949	1	**0.00032**	0.013	0.035	0.130	1	0.719	-0.033	0.017	4.228	1	**0.040**	0.036	0.012	8.384	1	**0.0038**
Elevation	0.00005	0.0004	0.038	1	0.845	0.002	0.001	5.514		**0.019**	0.001	0.001	0.562	1	0.454	0.002	0.001	3.042	1	0.081	0.001	0.001	3.060	1	0.080
Road Density	321.300	69.810	21.123	1	**4.3x10** ^**-6**^	644.100	130.400	34.293	1	**<10** ^**−6**^	-80.790	161.100	0.250	1	0.617	583.854	112.845	32.931	1	**<10** ^**−6**^	-894.300	85.230	133.941	1	**<10** ^**−6**^
Fire Hazard—High	-0.178	0.091	3.787	1	0.052	-0.103	0.243	0.176	1	0.675	0.375	0.250	2.403	1	0.121	0.110	0.193	0.327	1	0.567	-0.169	0.123	1.844	1	0.174
Cover Type			9.206	3	**0.027**			6.757	3	0.080			13.732	3	**0.0033**			5.600	3	0.133			3.246	3	0.355
Barren	0.056	0.208				-0.693	0.411				3.113	158.700				-0.648	0.304				0.166	0.331			
Forest	0.576	0.213				-0.664	0.523				3.352	158.700				0.639	0.413				0.727	0.465			
Herbaceous	-0.896	0.479				1.552	0.632				-11.080	476.100				-0.130	0.385				-1.156	0.836			
Shrub	0.264	0.202				-0.196	0.387				4.614	158.70				0.140	0.297				0.262	0.361			

Unburned structures tended to have higher sales price than burned structures (Fig 3I), a pattern that occurred in most sales years (Fig 2). Specifically, more than twice the fraction of structures were damaged among properties valued at less than $300,000 (0.203) compared to properties valued between $300-$600,000 (0.0819; Fig 3I). In a statistical model using data from all five fires (Table 3), burn damage decreased significantly with greater (natural log of) property price. Comparing a model that did not include property price (null model) to one that did, the one that included price explained more variation in the data (coefficient = -0.345, *χ*^2^ = 20.11, *df* = 1, *p* < 7.3 x 10^-6^). Similarly, a price-fire identity interaction was also statistically significant (*χ*^2^ = 65.597, *df* = 4, *p* < 10^-6^).

**Fig 4:**
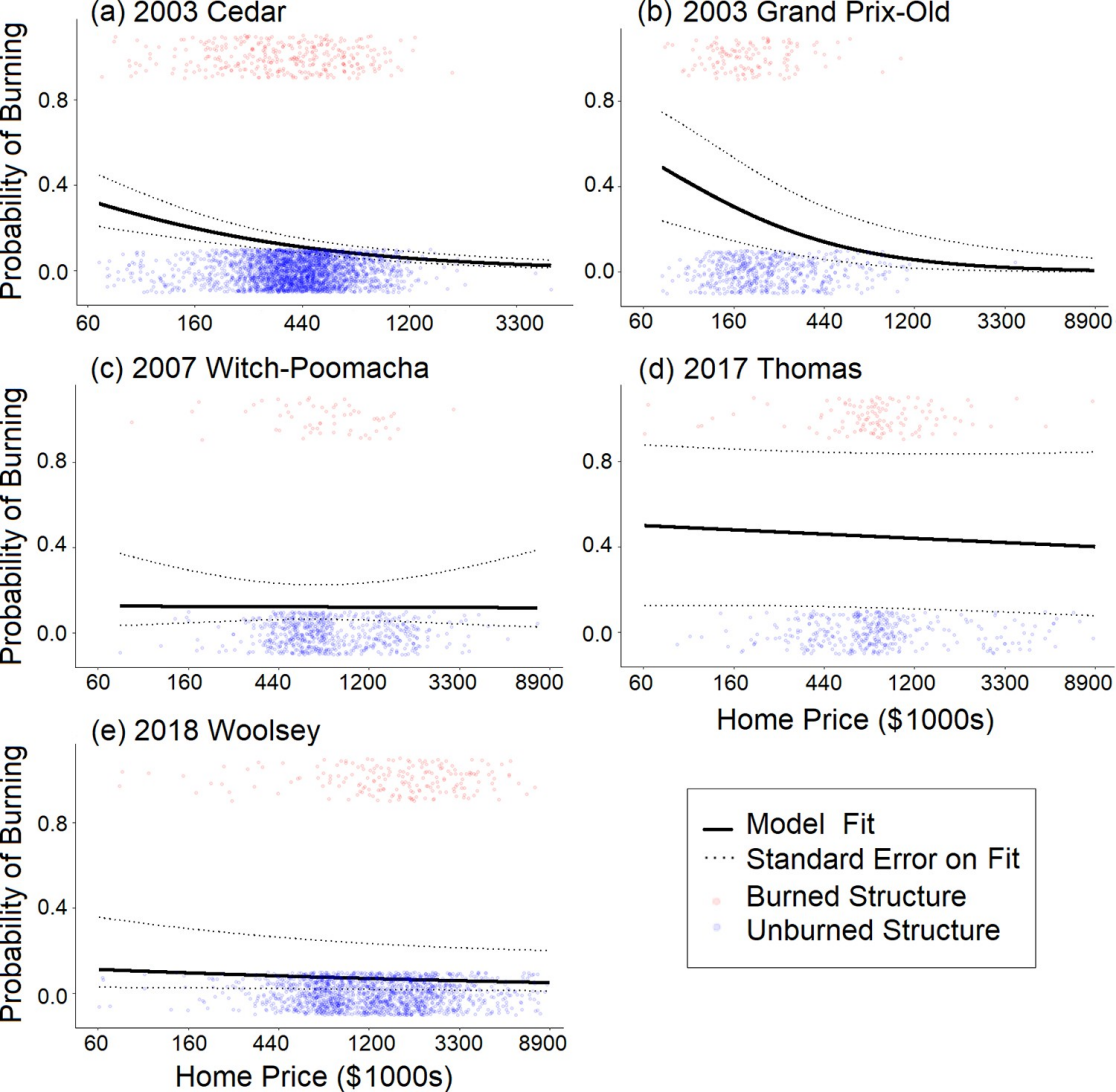
Model fit for the single-fire models. Black lines show model fit and dashed black lines show standard error in fit for each of the single-fire models. Blue points jittered around zero (to show point density) show unburned structure prices and red points jittered around one show burned structure prices.

When considering fires individually, structures within two out of five fires had a significantly lower likelihood of burn damage with increasing property price (negative model coefficients): Cedar (*χ*^2^ = 27.67, *df* = 1, *p* < 10^-6^) and the Grand Prix-Old complex (*χ*^2^ = 15.21, *df* = 1, *p* < 9.6 x 10^-5^; Table 4, Fig 4). Although subtle, Fig 1A and 1C shows smaller red (burned) than blue (unburned) points within these fires, where point size is scaled to price. Of the three fires without a significant relationship between the likelihood of structure damage and property price, all had negative coefficients.

Considering alternative subsets of data in Supplemental Information S1, we found that our results are robust to different inclusion criteria and the use of assessed value data. When we looked at all sales after 1996, the property price coefficient on the resulting 13-fire dataset was negative but not significant (S3 Table in S1 File). Looking at individual fires with this dataset, the Cedar and Grand Prix-Old Fires still had a significant negative relationship between property price and burn damage, as did the 2007 Grass Valley Fire (S4 Table in S1 File). When we looked at all sales during the recession (and only including sales within seven years before a fire), the coefficient on the resulting ten-fire dataset was negative and significant (S5 Table in S1 File). Looking at individual fires with this dataset, the Cedar and Grand Prix-Old fires also had a significant negative relationship between property price and burn damage, as did the 2008 Tea Fire (S6 Table in S1 File). Across these two analyses, there were no fires with a significant positive relationship between property price and burn damage. When we considered only sales that occurred within two years of the Cedar and Grand Prix-Old Fires–to reduce the potential for prices to diverge between the date of sale and the date of the wildfire–we again found that there was a significant negative relationship between property price and burn damage (S7 Table in S1 File). Finally, we considered assessed value data and found a significant overall negative relationship between assessed value and burn damage (S8 Table in S1 File) and significant negative relationships for the following fires when considered individually (S9 Table in S1 File): 2003 Cedar Fire, 2007 Rice Fire, 2008 Tea Fire, and 2018 Woolsey Fire. In all the subsets of data we explored across property sales price and assessed value, we found one fire–the 2007 Corral Fire–with a significant positive relationship between burn damage and assessed value (S9 Table in S1 File), but not property price and burn damage.

**Fig 5:**
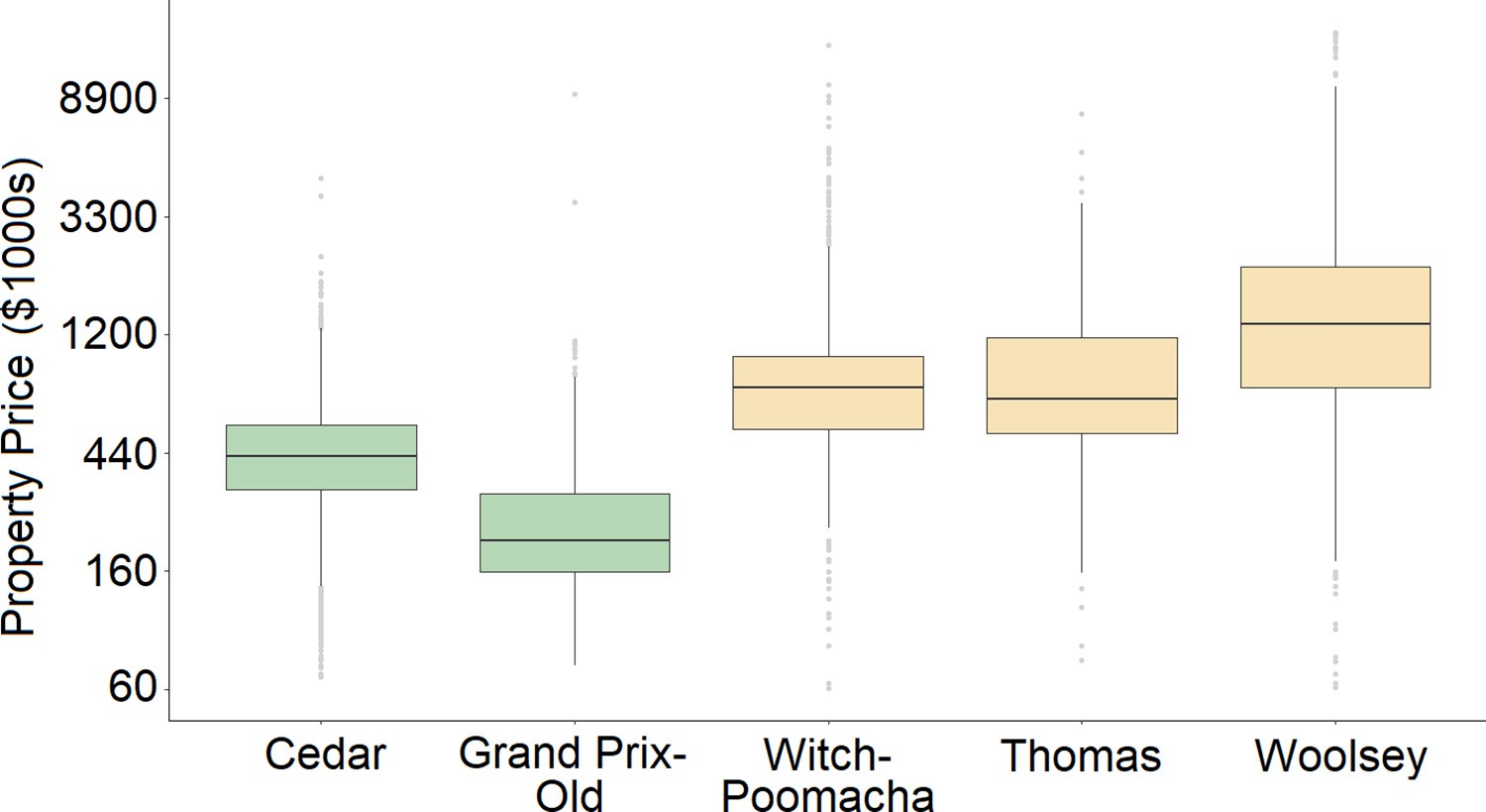
Boxplot of prices for individual fires. Boxes show interquartile range of property price, lines show median property price, and gray dots show outlier prices within each fire, where burned and unburned structures are combined. Boxes colored in green are for fires that showed a significant relationship between property price and structure damage. Boxes colored in yellow are for fires where there was no relationship.

Overall, the relationship between property price and burn damage was robust to different subsets of sales data for the Cedar and Grand Prix-Old Fires. The Cedar and the Grand Prix-Old Fires were two of the largest fires and two of the earliest fires in S1 Table in S1 File. Property values in Southern California increased substantially over our study period, as reflected in the distribution of property prices for each of the five fires (Figs 2 and 5). Given that the oldest fires in the dataset had a significant relationship between burn damage and property price, we explored the relationship between burn damage and year built in two datasets: one using sales data and the other assessed value. The sales dataset used the five-fire dataset presented here where observations on year built were available. This resulted in a much restricted dataset, especially for burned structures (S10 Table in S1 File). We found that year built was statistically significant in a model that considered a linear relationship between burn damage and year built (*χ*^2^ = 45.26, *df* = 1, *p* < 10^-6^; S11 Table in S1 File) and when year built was a factor of three distinct time windows: before building code legislation in 1991, between 1991–2008, and after building code legislation in 2008 (*χ*^2^ = 35.48, *df* = 2, *p* < 10^-6^; S12 Table in S1 File). Further, in these models that included year built, property price was significantly negatively related to burn damage (*χ*^2^ = 15.16, *df* = 1, *p* < 9.91 x 10^-5^; S11 Table in S1 File; and *χ*^2^ = 20.11, *df* = 1, *p* = 7.3x10^-6^; S12 Table in S1 File). Similar results–significant negative coefficients for year built and assessed value–were found for data using assessed values across nine fires (S13-S15 Tables in S1 File).

**Fig 6:**
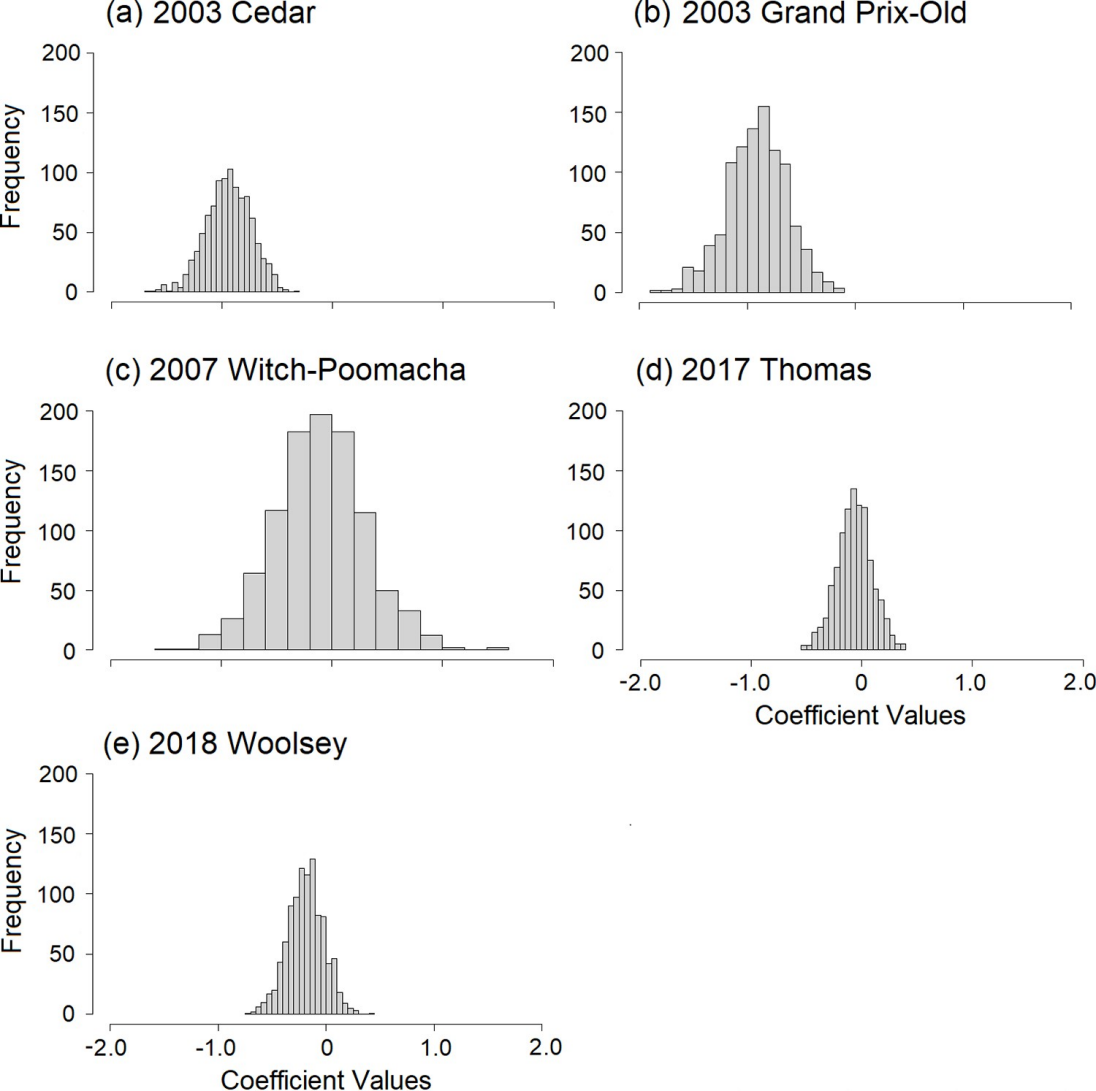
Histogram of property price model coefficients. Coefficients are shown across single-fire models using 1000 subsamples of data selected for each fire from the full dataset. Data subsets included an equal number of burned and unburned structures consistent with 0.9 times as many burned structures as were available for each fire perimeter.

Across fires, there were more unburned than burned structures and many high and low property price outliers (Fig 5). The combination of more unburned data with greater outliers could potentially inflate the statistical significance of logistic regressions. Thus, we ran logistic regressions on 1000 bootstrapped subsamples of data that included equal numbers of burned and unburned structures. Specifically, 300, 117, 50, 109, and 171 burned structures (or 0.9 times the number of burned structures within a fire perimeter) were used for the Cedar, Grand Prix-Old, Witch-Poomacha, Thomas, and Woolsey Fires, respectively. The resulting histograms of model coefficients (Fig 6) are consistent with Table 4. The Cedar and Grand Prix-Old Fires had negative property price coefficients for all of the 1000 models run on bootstrapped data. The Witch-Poomacha, Thomas, and Woolsey fires had 603, 665, and 876 negative coefficients out of 1000, respectively.

## Discussion

We found evidence that higher priced properties were less likely to burn across the most destructive fires in Southern California from 2000–2019. This was reflected in models that included all fires together, the two earliest (2003) individual fires, and models built on bootstrapped data. Specifically, our individual-fire models suggest that a $270,000 property had a 15.0% and 21.3% chance of damage in the Cedar and Grand Prix-Old fires, respectively (assuming all other model covariates and factors were held at their mean and median values, respectively). Whereas, a $730,000 property had an 8.2% and 9.2% chance of being damaged (log-odds ratio coefficients for log-price are -0.68 and -0.98 for the Cedar and Grand Prix-Old Fires, respectively).

We initially hypothesized that structure damage would be lower for higher-priced properties because owners of more valuable properties are likely to have more resources to dedicate to home and neighborhood protection, with implications to equity [16]. Previous studies have found that household income and total wealth significantly increase the likelihood that a homeowner will make structural modifications to increase wildfire protection [18, 19]. These may be sound investments, as research shows that these modifications can be effective at reducing wildfire damage [29, 33, 46]. Pre-fire home modifications may also benefit suppression efforts, which is what Plantinga et al. [47] hypothesized after finding more effective wildfire suppression on the edges of wealthier neighborhoods. While the Zillow dataset includes information on roof type and building construction, there were insufficient observations to allow for their inclusion in this analysis. Thus, we can only hypothesize the potential influence of better homeowner mitigation practices in higher-priced properties.

The location of the Grand Prix-Old Fire may help explain why this fire had a significant relationship between property price and structure damage. The Grand Prix-Old Complex occurred farther inland, had the lowest property price (Fig 5), and had the highest level of neighborhood poverty based on census tract data (Table 5). Inland properties in Southern California tend to be of lower value and in neighborhoods with increasing poverty, compared to more coastal neighborhoods with greater affluence [48]. Census data also show that the Grand-Prix-Old Complex was majority non-white with high asthma rates (Table 5), suggesting that this fire impacted a more vulnerable community. The census data suggest that the Cedar Fire, despite occurring farther inland compared to the Woolsey and Thomas fires, did not occur in disadvantaged neighborhoods. However, both the Grand Prix-Old and the Cedar Fires had the biggest difference in neighborhood poverty between burned and unburned properties (Table 5). This suggests that both property owner wealth and neighborhood wealth may contribute to a structure’s likelihood of burning.

**Table 5:** Demographic data underlying burned and unburned structures. Demographic data come from CalEnviroScreen 4.0 which summarizes U.S. Census data at the resolution of the census tract. Values in the table are the means ± standard deviations across structures within a given fire perimeter.

Fire Name	Number of Census Tracts Within Fire Perimeter	% of Population that is White		% of Population >64 or <10 Years of Age		% of Population Living At or Below Twice the Poverty Level		Number of Asthma-related Emergency Room Visits per 10,000 Residents^1^	
		Unburned	Burned	Unburned	Burned	Unburned	Burned	Unburned	Burned
Cedar	26	78.2 ± 8.1	74.8 ± 10.0	72.2 ± 3.4	68.6 ± 4.5	12.8 ± 6.6	18.4 ± 6.4	24.8 ± 7.2	24.1 ± 10.2
Grand Prix-Old	23	35.4 ± 18.8	34.7 ± 21.0	74.5 ± 2.9	73.6 ± 2.2	31.6 ± 12.6	37.2 ± 6.9	82.4 ± 38.8	101.1 ± 32.3
Witch-Poomacha	20	67.6 ± 14.0	75.8 ± 10.1	67.7 ± 7.1	63.3 ± 9.5	14.1 ± 5.4	15.7 ± 6.0	21.3 ± 7.7	22.3 ± 8.1
Thomas	19	66.8 ± 17.7	72.3 ± 11.6	65.3 ± 5.7	64.8 ± 4.1	16.1 ± 7.1	13.6 ± 6.3	34.7 ± 8.3	37.8 ± 7.4
Woolsey	30	74.5 ± 9.2	77.6 ± 8.1	71.2 ± 6.1	70.2 ± 6.0	11.8 ± 4.3	11.9 ± 1.6	18.8 ± 7.1	13.3 ± 2.5

^1^ Across California census tracts, the 50^th^ percentile for asthma-related emergency room visits is 45.7 visits per 10,000 residents.

In addition to the potential influence of owner wealth on fire hardening, there may also be an influence of owner knowledge about wildfire preparedness [49]. The earliest fires showed the strongest influence of property price on burn probability. Through time, all property owners–even the most resource-poor–may take more fire protective measures as they witness increasing wildfire damage in Southern California neighborhoods (similar to findings in [19, 22]). Further, new building codes were introduced in 2008 to require fire resistant materials in roofing, decking, exterior wall, and vents. If greater adoption of fire hardening through time has led to more homogeneity in property preparedness across the later fires, this could also explain why later fires did not show a relationship between property price and structure damage. Further, other studies looking at wildfire exposure in the latter half of our study period found that higher-priced homes may be more exposed to wildfire. Specifically, looking at wildfires from 2011–2018 across the Western US, Wibbenmeyer and Robertson [50] found that the highest priced homes were more likely to be in areas of high wildfire risk and within fire perimeters.

Previous studies have found that the year a structure was built can have a dramatic influence on the ability to withstand wildfire exposure. Baylis and Boomhower [32] found that a home built in California after the 2008 adoption of new building codes was 40% less likely to sustain wildfire damage than a home built in 1990; further, there was not a dramatic 2008 decrease in wildfire damage in states that had not adopted new building codes. We explored year built for our dataset in Supplemental Information S1 (S10-S15 Tables in S1 File). Unfortunately, many of the structures damaged in the earlier fires were rebuilt and the Zillow dataset only reported the year of reconstruction. Removing all of the reconstruction observations (observations where year built was listed as after the fire) resulted in a loss of more than 50% of observations of burned structures. Further, the remaining burned structures were shifted to higher price compared to the original distribution of property prices for burned structures. Still, our analysis showed a significant negative relationship between property price and burn damage. We also found a significant negative relationship between year built and burn damage (S11-S15 Tables in S1 File), in support of [32].

Similar to previous studies (e.g. [23, 25]), we found landscape and topographic covariates influenced the likelihood of structure damage. However, the influence of variables depended on the scale of the analysis, which is consistent with [26] and [31]. Elevation and driving time to fire station were significant in the five-fire model but only one single-fire model: elevation was significant in the Grand Prix-Old Complex individual model and driving time in the Cedar model. The significance of these variables is likely due to the Grand Prix-Old and Cedar Fires having the broadest ranges of elevation and driving time values, respectively. To our knowledge, driving time to fire station has not been explored in previous models describing structure damage and loss. However, when there are multiple simultaneous fires, driving time becomes less important because local suppression resources are typically overwhelmed and personnel and engines may come from distant stations. Previous studies have suggested that housing arrangement might influence firefighter accessibility and thus the likelihood of burn damage [31]. Further, increased damage has been observed in structures that were farther from communities that had previously done wildfire planning [34]. Finally, vegetation type was important in our models, but less important in previous models [31]. Interestingly, the Cedar, Grand Prix-Old, Tea, and Grass Valley Fires tended to have more forested areas within the fire perimeter and in the areas surrounding structures.

In our full model, road density was surprisingly not a good predictor of structure damage. Previous work has suggested that the highest fire densities typically occur at intermediate population [1] and structure densities [51]. Expecting road density to be correlated with population density or structure density, we were surprised that structure damage was lower for properties with road densities in the middle of the observed range (Fig 2). We tried a quadratic term in the model but it was not significant. Moving to single-fire models, the effect of road density was significant (Table 4) in four out of five fires (the Witch-Poomacha Complex was the exception) and was positive (greater likelihood of burning with increasing road density) in three out of four of the significant models (Woolsey was the exception). Each fire had a similar range of road density values compared to the five-fire dataset; it is not immediately clear why road density was significant primarily in local, single-fire models.

Because destructive wildfires are infrequent events, studies of wildfire exposure and damage are often dictated by only a few fires [50]. We focused our study on Southern California, a region that has experienced repeated wildfires resulting in structure loss over the past two decades. Still, there were few fires that met our requirement of having 25 burned structures with available Zillow property sales data. The recession further limited the availability of property price data for this study because we did not include any sales that occurred within the pre-recessionary housing bubble (2004–2007), the recession (2007–2009), and post recessionary recovery (2010–2012). By relaxing our inclusion criteria and using assessed value data in analyses in Supplemental Information S1, we were able to explore an additional ten wildfires, making up two-thirds of wildfires in Southern California with >100 structures damaged or destroyed during 2000–2019. Doing so, we found four more fires with statistically significantly increasing likelihood of burn damage with decreasing price or assessed value: the 2007 Grass Valley Fire, 2007 Rice Fire, 2008 Tea Fire, and 2018 Woolsey Fire (S4, S6, and S9 Tables in S1 File). We also found one fire, the 2007 Corral Fire, that had increasing burn damage with increasing assessed value (S9 Table in S1 File). We did not emphasize these additional fires because the relationship between property value and burn damage was not robust to data inclusion criteria. For example, the significant relationships between property value and burn damage seen in the Woolsey and Corral Fires were only present with the assessed value data and not the property price data. However, taken together, the results in Supplemental Information S1 increase the robustness of our results by providing additional evidence suggesting an overall relationship of increasing burn damage with decreasing property value. Still, additional studies over larger spatial extent and studies that model property values for all burned and unburned structures will be important to determine whether our results are broadly applicable.

This paper explored the relationship between property price and structure damage within properties that were exposed to wildfire. It is also possible that lower priced properties and less affluent neighborhoods are disproportionately exposed to wildfire in the first place. Using census tract data from 1999–2015 and assessor data from 2011–2015, Plantinga et al. [47] found that, after controlling for environmental covariates that influence wildfire spread, wildfire suppression activities were more likely to stop the spread of wildfire into wealthier neighborhoods. While ground-based wildfire suppression activities–in the form of hand crews and engines–increase as a function of the number of threatened structures within a neighborhood, regardless of neighborhood affluence, more affluent neighborhoods were more likely to receive aircraft support [52].

Future studies should extend the work presented here with high-resolution comparisons of locations that burned just inside versus outside a fire perimeter to see if there is a relationship between property value and wildfire exposure. Other studies have approached this using less resolved census-tract data [13, 14, 47, 48, 53], which does not have the same resolution as the Zillow data. Similarly, future studies should consider the influence of explicit hedonic variables to burn damage. The fact that we saw a significant property-price fire identity interaction suggests that neighborhood characteristics are likely important to the likelihood of burn damage and loss. Future studies might also consider building a model to estimate property price across all structures within and surrounding fire perimeters. Such a model would allow for explicit consideration of neighborhood characteristics and could incorporate a larger number of observations with greater representation of structural features (e.g. older structures). For example, the fact that we limited our analysis to sales directly preceding a fire means that we likely removed lower value properties that are less likely to be readily bought and sold. Thus, our analysis likely underestimates the relationship between burn damage and both property value and year built. A price model could provide a more nuanced understanding of these specifics.

As far as we know, our study is among the first to explore the relationship between property price and the likelihood of structural damage during wildfire. However, there have been numerous studies on the importance of wildfire on subsequent property price. An immediate, but short-lived decrease in home price followed fires in Colorado [54] and California [55]. Further, the impacts of wildfire and post-wildfire flooding can interact to lower home prices as a function of proximity to these disasters [56]. These studies suggest that even if homes are not damaged in a wildfire, homeowners may still suffer a loss in property value following a fire. Thus, if less affluent communities really do experience higher exposure to wildfire [13, 14, 48], this could have even further impacts on less affluent homeowners. On the other hand, national trends showing overall migration from rural areas in the Midwest to fire-prone locations in the West suggest that wildfire risk is not the most influential predictor of where Americans are choosing to live [57].

Consistent with recommendations of Auer [16], our results suggest a holistic, multijurisdictional approach to wildfire management that accounts for potential disproportionate impacts of wildfire damage. To achieve this, more research is needed to quantify the generalizability or limits to our findings and the potential mechanisms leading to increasing wildfire damage with increasing social vulnerability. A mechanistic understanding of the interaction between social vulnerability and wildfire vulnerability would inform which resources could best benefit less affluent neighborhoods and homeowners. Meanwhile, we recommend decision makers consider explicitly distributing resources to the most socially vulnerable communities within wildfire-prone locations. Part of this conversation should also include considerations about where to build. Leapfrog development, as opposed to infill, has been shown to lead to considerably greater wildfire risk to structures [51]. Further, land use planning that prioritizes decreasing wildfire risk has the potential to benefit conservation of natural habitat in Southern California [58], a biodiversity hotspot of continental importance [59].

## Conclusions

We found evidence of increasing burn damage with decreasing property price in Southern California, with the strongest relationship in older and inland fires. These results were robust to analyses run on alternative subsets of the data and bootstrapped data. Consistent with other studies, we found that landscape characteristics were also important to the likelihood of burn damage. To advance equitable prioritization of resources to promote wildfire risk mitigation, further research is critically needed to determine why less valuable properties might experience greater damage.

## Supporting information

S1 File (DOCX)
