# Supplementary material for: Evidence of increasing wildfire damage with decreasing property price in Southern California fires
Source: PLoS One. 2024 Apr 24;19(4):e0300346. doi: 10.1371/journal.pone.0300346 (PMC11042721; doi:10.1371/journal.pone.0300346)
Supplement: S1 File — (DOCX) [file pone.0300346.s001.docx]

**Supplemental Information S1 for:**

**“Evidence of increasing wildfire damage with decreasing property price in Southern California fires”**

Erin Conlisk, Van Butsic, Alexandra D. Syphard, Sam Evans, Megan Jennings

Analyses Using Alternative Inclusion Criteria

We started with a given number of digitized burned structures and unburned structures for 2000-2010 from the appendix of Alexandre et al. (2016). We augmented this data with remotely sensed observations of burned structures from 2011-2019 (Syphard et al. 2019). S1 Table shows the number of observations available and the number of structures destroyed (according to CALFIRE incident reports) for the most destructive fires in Southern California from 2000-2019. For a variety of reasons (poorly resolved images, vegetation cover, multiple structures on a property, etc.), only 37% of structures that were reported destroyed in CALFIRE incident reports corresponded to unique property parcels in the remotely sensed data.

Starting with these remotely sensed data, we analyzed five fires in the main paper with structures that corresponded to sales data consistent with the following data inclusion criteria. First, we included structures that had been sold within seven years preceding a fire. Seven years was chosen as a compromise between retaining only recent sales and having enough observations for analysis. Second, we removed sales during the period of 2004-2012 because we saw evidence that the housing bubble and post-recession depreciation affected less valuable properties in inland neighborhoods more than valuable properties in coastal neighborhoods. Next, we eliminated properties with sales for less than $50,000 and greater than $15,000,000 and properties with non-single-family, residential use codes. We removed any observations where year built (likely representing a major remodel) was after the sale date. Finally, to include the observations within a given fire perimeter, we required at least 25 burned and unburned structures within the perimeter. Applying these criteria, we were left with five fires that we explored in the main paper. According to CALFIRE incident reports from 2000-2019, each of these fires had at least 1,000 structures destroyed within their perimeters (Table 1). The fraction of burned structures with available sales data hovered around 11-15% for all fires except the Witch-Poomacha complex, where only 3% of structures destroyed had available data. (The Witch-Poomacha complex occurred in 2007 and thus removing sales during the real estate bubble of 2004-2008 reduced the fraction of available data.)

To explore the robustness of our results using sales data on the five-fire dataset in the main paper, each of the above criteria were strengthened or relaxed to remove or include fires. Allowing fires with as few as 15 burned structures to be analyzed, we analyzed three modified sales datasets with the following criteria: (i) including any property sales occurring after April 1996 (the date that, according to the ZTRAX FAQ website (2018), most counties have robust sales data), but not including data from 2004-2012; hereafter the “Early Sales” dataset; (ii) including property sales during the real estate bubble and recession (and within the seven years preceding a fire), or the “Includes Recession” dataset; and (iii) including property sales within only two years before a fire, the “Two-Year Window” dataset.

In addition to relaxing these inclusion criteria, we also explored assessed value data because some of the fires that could not be explored with the sales data could be explored with assessed value. We analyzed an additional dataset using assessor data from 1996 onward. We kept assessed value data from the real estate bubble and recession, observing that assessor data was less influenced by the bubble and recession. We eliminated properties with assessed values less than $50,000 and greater than $15,000,000; eliminated properties with non-single-family, residential use codes; removed any observations where year built was after the transaction date; and kept fires with as few as 15 burned structures. We used the Recording Date listed in the ZTRAX assessor data to estimate the date of the property assessment (usually assigned at the time the property was sold). We found a linear trend between assessed value and the year of the Recording Date; thus, we included Recording Date as a variable in the model. We understand that assessed value can often diverge from sales data given that California Proposition 13 limits the annual growth in property value to 2%. Of the observations with assessed value data (and with sales prices greater than $50,000 and less than $15,000,000), we found a correlation coefficient between the assessor data and the transaction data of *r =* 0.65.

Ultimately, our analysis was limited by the number of destructive fires in Southern California across 2000-2019. Given that we observed a maximum of 15% of damaged properties having been sold within the seven years preceding a fire (Table 1), we would expect that only fires with greater than 100 structures destroyed might be eligible for inclusion in our extended analyses. Within the study area and study period of 2000-2019, there were 22 fires with at least 100 structures destroyed within fire perimeters (CALFIRE incident reports from 2000-2019). With either sales or assessed value data, we were able to analyze 15 out of 22 fires with more than 100 burned structures. The “Early Sales” dataset allows us to explore 13 fires (S2 Table), including one fire (Corral) with fewer than 100 total burned structures reported by CALFIRE. S1 Table shows that there were an additional ten fires with at least 100 structures destroyed that did not have enough sales data to be included. Using the assessed value data, we were able to explore an additional three fires, for a total of 16 fires (15 with greater than 100 destroyed structures). Thus, while 16 fires may not seem like many, the analyses included in this Appendix explored the effect of price or assessed value on the likelihood of burn damage for over two-thirds of the potentially eligible Southern California fires from 2000-2019. Further, as compared to other sources of information, CALFIRE tends to list more destroyed structures. Thus, we are likely *overestimating* the number of potentially eligible fires in S1 Table. Looking at the number of observations across fires, we were able to retain a maximum of 20% (using sales data) and 38% (using assessed value data) of individual burned structure observations identified by remotely sensed data. (Percentages were obtained by comparing the sum of structures in either the Early Sales or Assessed Value data in S2 Table to the sum across the available remotely sensed burned structure data in S1 Table.)

Overall, the additional analyses presented here agree with the analyses in the main paper, with the strongest differences seen in the Early Sales and the Assessed Value datasets. While the coefficient for property price is still negative in the Early Sales dataset, it is no longer significant. The remaining variables largely have the same relationship as the main model (comparing S3 Table and Table 3). Given a greater number of potential years in which sales can occur, sales year becomes significant (*χ*^2^ = 4.497, *df* = 1, *p* = 0.034) in the Early Sales dataset (S3 Table). The interaction between price and the identity of the fire is significant (*χ*^2^ = 101.76, *df* = 12, *p* < 10^-6^), prompting us to explore each fire individually. Of the 13 fires analyzed, four had positive coefficients (Witch-Poomacha, Slide, Freeway, and Corral Fires), but none of these were significant. Two fires had marginally significant negative coefficients: the Tea (*χ*^2^ = 3.718, *df* = 1, *p* = 0.054) and Harris Fires (*χ*^2^ = 2.981, *df* = 1, *p* = 0.084). Three fires (Cedar, Grand Prix-Old, and Grass Valley Fires) had significant negative coefficients (S4 Table; where Table 4 only includes fires with a significant relationship between price and the likelihood of burn damage). While the main paper and the remainder of Supplemental Information S1 will show that the relationship between property price and likelihood of burn damage is robust for the Cedar and Grand Prix-Old Fires, the coefficient for the Grass Valley Fire is highly sensitive to minor omissions of observations. The Grass Valley Fire no longer has a significant effect of price, or even marginally significant, if the lowest priced burned property is removed from the analysis.

S5 Table shows results for models that include sales from the bubble and recession period. Like the results in the main paper (Table 3), the overall coefficient for price is significant (*χ*^2^ = 8.52, *df* = 1, *p* = 0.0035; S5 Table). There was a significant interaction between price and fire identity (*χ*^2^ = 76.08, *df* = 9, *p* <10^-6^). Exploring the ten fires in the Includes Recession dataset individually, seven fires had negative coefficients for price, three of which were significant: Cedar (*χ*^2^ = 27.68, *df* = 1, *p* < 10^-6^; S6 Table), Grand Prix-Old (*χ*^2^ = 15.22, *df* = 1, *p* = 9.6x10^-5^), and Tea (*χ*^2^ = 9.60, *df* = 1, *p* = 0.0019).

Taken together, including more fires in the analysis – either by including sales as far back as 1996, or including data from the recession (S3-S6 Tables) – leads to a similar conclusion as the main paper: there is evidence for a negative relationship between property price and wildfire damage. Further, the negative relationship between price and likelihood of burn damage is robust for the Cedar and Grand Prix-Old Fires. In addition to the Early Sales and Includes Recession datasets, the Cedar and Grand Prix-Old Fires have a negative relationship between price and burn damage in the Two-Year-Window subsample of data (*χ*^2^ = 22.11, *df* = 1, *p* = 2.6x10^-6^ and *χ*^2^ = 11.06, *df* = 1, *p* = 0.00088; S7 Table), where the Two-Year-Window dataset only included sales within two years of a fire (with sample sizes shown in S2 Table). The Cedar and Grand Prix-Old Fires were the only fires that have enough data to analyze within a two-year time window.

While sales price differs from assessed value, there were more structures that had an assessed value as compared to a sales price. Thus, we included a model that looked at the probability of burn damage as a function of the total assessed value (S8 Table). Again, we found a significant negative overall relationship between assessed value and likelihood of burn damage (*χ*^2^ = 11.41, *df* = 1, *p* =0.00073; S8 Table). All environmental variables were also significant and there was a significant interaction between price and fire identity (*χ*^2^ = 33.77, *df* = 12, *p* = 0.00073). Exploring fires individually, we found the biggest differences from the model in the main paper. In particular, the Grand Prix-Old Fire no longer had a significant relationship between assessed value and burn damage (although there was a marginally significant relationship; *χ*^2^ = 2.900, *df* = 1, *p* = 0.089). Four fires had a significant negative relationship between assessed value and burn damage: Cedar (*χ*^2^ = 7.73, *df* = 1, *p* = 0.0054), Rice (*χ*^2^ = 11.52, *df* = 1, *p* = 0.00069), Tea (*χ*^2^ = 7.89, *df* = 1, *p* = 0.0050), and Woolsey (*χ*^2^ = 13.78, *df* = 1, *p* = 0.00020). With this dataset, there was one fire, the Corral Fire, with a significant positive relationship between assessed value and probability of burn damage (*χ*^2^ = 4.61, *df* = 1, *p* = 0.032). Across all subsets of data, the Corral Fire was the only fire with a significant positive relationship between assessed value or sales price and burn damage. Further, the positive relationship seen in the Corral Fire was not significant when property price was used instead of assessed value.


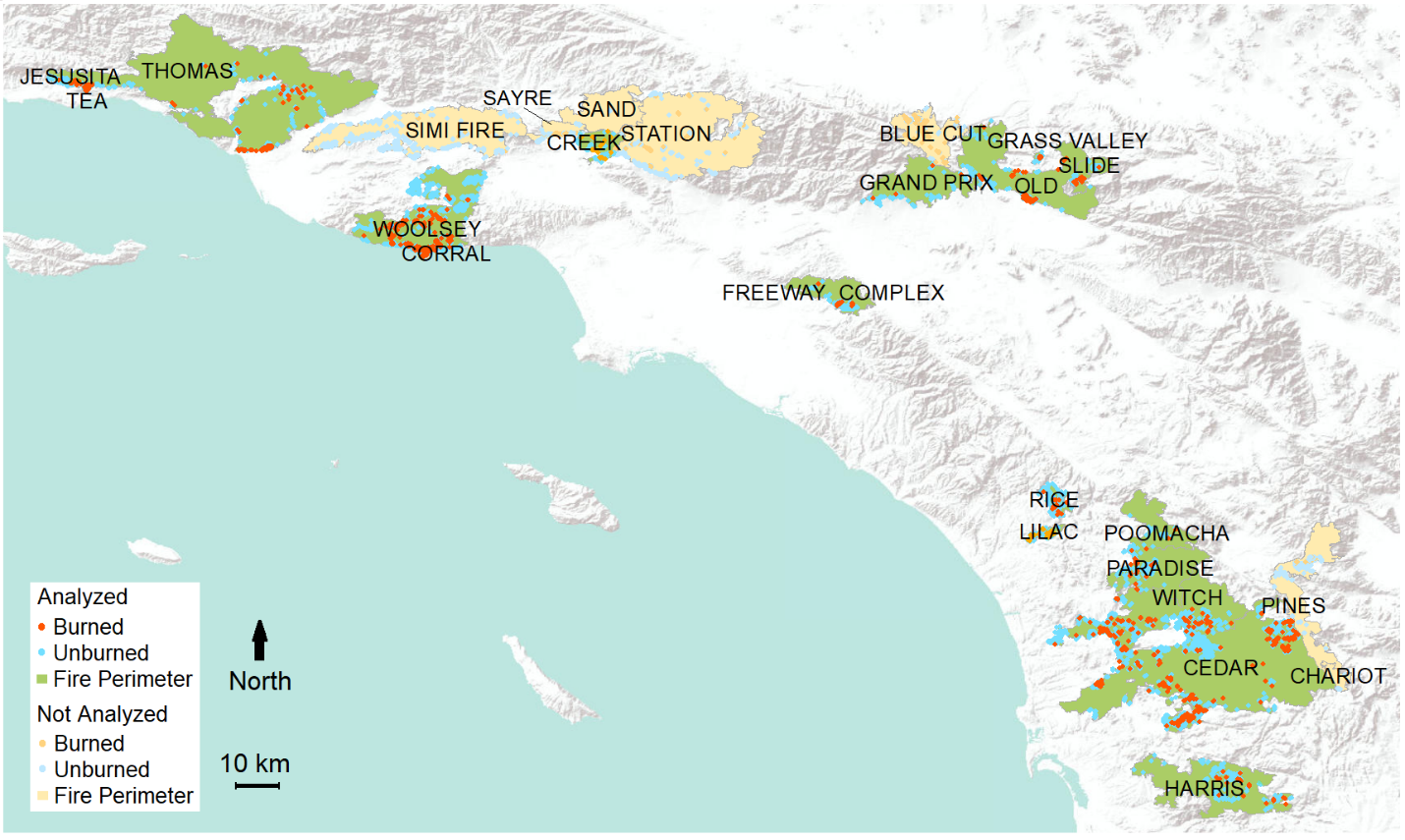


**S1 Fig. Map of wildfires analyzed in the Supplemental Information.** Map includes analyzed wildfires (listed in S2 Table) and wildfires with greater than 100 burned structures but not enough sales data to be analyzed (listed in S1 Table). The darker “analyzed” points were used in the “Early Sales” dataset – thus each point has a property value associated with it. The lighter “Not Analyzed” points represent all the structure data, regardless of whether there was a sale associated with the property. Topographic base map provided by ESRI (2023).

**S1 Table. Summary table of the number of burned and unburned properties.** The first set of columns in the table lists the number of burned and unburned properties obtained through remote sensing, before checking for the availability of property sales data or assessed value data for fires with >100 burned structures (and the Corral fire which had enough sales data to be included in analyses). The next column lists the number of structures reported destroyed by CALFIRE. The last column lists whether the fire was used in an analysis. Table S2 shows the number of observations available after incorporating property value information.

| **Fire Name** | **Year** | **Burned Properties Available in Remotely Sensed Dataset** | **Unburned Properties Available in Remotely Sensed Dataset** | **CALFIRE reported structures destroyed** | **Included in Analyses** |
| --- | --- | --- | --- | --- | --- |
| Pines | 2002 | 17 | 249 | 160^1^ | No |
| Cedar | 2003 | 1384 | 7351 | 4847^1^ | Sales/Assessor |
| Grand Prix-Old | 2003 | 442 | 1869 | 1139 | Sales/Assessor |
| Paradise | 2003 | 77 | 722 | 223 | Sales/Assessor |
| Simi | 2003 | 16 | 1342 | 300 | No |
| Witch-Poomacha | 2007 | 529 | 5013 | 1867 | Sales/Assessor |
| Corral | 2007 | 55 | 223 | 86 | Sales/Assessor |
| Grass Valley | 2007 | 94 | 267 | 178 | Sales |
| Harris | 2007 | 110 | 731 | 472 | Sales/Assessor |
| Rice | 2007 | 67 | 1007 | 248^1^ | Sales/Assessor |
| Slide | 2007 | 80 | 1044 | 272 | Sales |
| Freeway | 2008 | 48 | 2563 | 200 | Sales |
| Sayre/Sylmar | 2008 | 518 | 439 | 634^1,2^ | No |
| Tea | 2008 | 208 | 193 | 210 | Sales/Assessor |
| Jesusita | 2009 | 39 | 281 | 160 | Assessor |
| Station | 2009 | 44 | 236 | 209^1^ | No |
| Chariot | 2013 | 0 | 45 | 149 | No |
| Bluecut | 2016 | 25 | 148 | 321^3^ | No |
| Sand | 2016 | 0 | 224 | 116 | No |
| Thomas | 2017 | 669 | 2702 | 1063 | Sales/Assessor |
| Creek | 2017 | 67 | 525 | 123 | Assessor |
| Lilac | 2017 | 157 | 1002 | 157 | Assessor |
| Woolsey | 2018 | 926 | 8167 | 1643 | Sales/Assessor |

^1^ The numbers of destroyed structures vary somewhat across news media, publications, and incident reports. Part of the discrepancy lies in what exactly is being reported (e.g. destroyed *structures* or destroyed *homes*). We used archived CALFIRE incident reports which typically reported higher values. For example, according to a UC ANR report (<https://ucanr.edu/sites/SAFELandscapes/files/79451.pdf>), the Pines, Sayre, and Station fires were reported to have only 37, 600, and 53 structures lost, respectively. Also, the Cedar fire was reported to have resulted in 2232 *homes* (not structures) destroyed (htps://news.caloes.ca.gov/looking-back-on-the-cedar-fire-20-years-later/).

^2^ The vast majority of structures destroyed in the Sayre/Sylmar Fire were mobile homes, where mobile homes are less likely to be recorded in the Zillow dataset.

^3^ Most of the structures destroyed in the Bluecut fire perimeter lay outside our study region.

**S2 Table. Summary of available data by fire for three different sales datasets and one assessed value dataset.** Datasets using sales data include: (i) “Early Sales” dataset which includes any property sales after April 1996; (ii) “Includes Recession” dataset which includes property sales during the real estate bubble and recession; and (iii) “Two-Year” dataset which includes property sales occurring within the two years before a fire. The Early Sales dataset includes thirteen fires with available assessed value data. All datasets included fires that surrounded as few as 15 burned structures.

| **Fire Name** | **Year of Fire** | **Number of Unburned Structures with Sales Data** | | | **Number of Burned Structures with Sales Data** | | | **Number of Unburned Structures with Assessed Value** | **Number of Burned Structures with Assessed Value** |
| --- | --- | --- | --- | --- | --- | --- | --- | --- | --- |
|  |  | **Early Sales** | **Includes Recession** | **Two-Year Window** | **Early Sales** | **Includes Recession** | **Two-Year Window** |  |  |
| Cedar | 2003 | 2827 | 2713 | 540 | 356 | 333 | 44 | 1704 | 269 |
| Grand Prix - Old | 2003 | 595 | 571 | 145 | 141 | 130 | 30 | 72 | 72 |
| Paradise | 2003 | 220 | 204 | ^1^ | 19 | 18 | ^1^ | 158 | 19 |
| Witch-Poomacha | 2007 | 1343 | 1432 | ^1^ | 147 | 146 | ^1^ | 2158 | 212 |
| Harris | 2007 | 148 | 166 | ^1^ | 23 | 27 | ^1^ | 291 | 43 |
| Slide | 2007 | 220 | 350 | ^1^ | 22 | 32 | ^1^ | ^1^ | ^1^ |
| Grass Valley | 2007 | 89 | 127 | ^1^ | 19 | 47 | ^1^ | ^1^ | ^1^ |
| Rice | 2007 | 276 | ^1^ | ^1^ | 15 | ^1^ | ^1^ | 446 | 23 |
| Corral | 2007 | 68 | ^1^ | ^1^ | 17 | ^1^ | ^1^ | 118 | 33 |
| Freeway | 2008 | 915 | ^1^ | ^1^ | 26 | ^1^ | ^1^ | ^1^ | ^1^ |
| Tea | 2008 | 22 | 12 | ^1^ | 29 | 18 | ^1^ | 130 | 144 |
| Jesusita | 2009 | ^1^ | ^1^ | ^1^ | ^1^ | ^1^ | ^1^ | 195 | 23 |
| Thomas | 2017 | 373 | 416 | ^1^ | 121 | 134 | ^1^ | 1471 | 499 |
| Creek | 2017 | ^1^ | ^1^ | ^1^ | ^1^ | ^1^ | ^1^ | 321 | 37 |
| Lilac | 2017 | ^1^ | ^1^ | ^1^ | ^1^ | ^1^ | ^1^ | 233 | 37 |
| Woolsey | 2018 | 1654 | 1654 | ^1^ | 190 | 190 | ^1^ | 5691 | 684 |

^1^ There were not enough points to analyze this fire in the specified analysis.

**S3 Table. Coefficient estimates and likelihood ratio test p-values for the Early Sales dataset.** Residual deviance was 6248 on 9839 degrees of freedom, with pseudo-r^2^ = 0.108.

|  | **Coefficient Estimate** | **Coefficient Std. Error** | **LR Chisq** | **Df** | **Pr(>Chisq)** |
| --- | --- | --- | --- | --- | --- |
| **Intercept** | -1.265 | 1.568 |  |  |  |
| ln(Price) | -0.103 | 0.121 | 0.734 | 1 | 0.392 |
| **Fire Identity** |  |  | 105.21 | 12 | <**10^-6^** |
| Cedar | 6.357 | 2.024 |  |  |  |
| Corral | -8.080 | 6.581 |  |  |  |
| Freeway | -27.370 | 7.240 |  |  |  |
| Grand Prix – Old | 14.670 | 2.766 |  |  |  |
| Grass Valley | 6.916 | 9.619 |  |  |  |
| Harris | 6.113 | 4.349 |  |  |  |
| Paradise | 0.412 | 5.178 |  |  |  |
| Rice | 4.603 | 6.230 |  |  |  |
| Slide | -10.510 | 4.946 |  |  |  |
| Tea | 13.170 | 6.909 |  |  |  |
| Thomas | 2.855 | 2.380 |  |  |  |
| Witch-Poomacha | -2.154 | 2.264 |  |  |  |
| Woolsey | -6.987 | 2.169 |  |  |  |
| **Station Distance (mins driving)** | 0.077 | 0.010 | 59.304 | 1 | <**10^-6^** |
| **Topographic Position Index (500m)** | 0.012 | 0.002 | 51.392 | 1 | <**10^-6^** |
| **Sales Year** | -0.036 | 0.017 | 4.497 | 1 | **0.034** |
| **Slope in 500m moving window** | 0.046 | 0.005 | 69.244 | 1 | <**10^-6^** |
| **Elevation** | 0.0004 | 0.0002 | 4.354 | 1 | **0.037** |
| Road Density (km/km2) | 16.300 | 25.550 | 0.407 | 1 | 0.524 |
| **Fire Hazard – High** | -0.128 | 0.050 | 6.579 | 1 | **0.010** |
| **Cover Type** |  |  | 21.177 | 3 | **9.7x10^-5^** |
| Urban, Ag, Barren, Water | -0.243 | 0.089 |  |  |  |
| Forest | 0.168 | 0.114 |  |  |  |
| Herbaceous | -0.191 | 0.164 |  |  |  |
| Shrub | 0.266 | 0.089 |  |  |  |
| **ln(Price) x (Fire Identity)** |  |  | 101.758 | 12 | <**10^-6^** |
| Cedar | -0.481 | 0.156 |  |  |  |
| Corral | 0.566 | 0.484 |  |  |  |
| Freeway | 1.923 | 0.529 |  |  |  |
| Grand Prix – Old | -1.129 | 0.221 |  |  |  |
| Grass Valley | -0.526 | 0.755 |  |  |  |
| Harris | -0.479 | 0.342 |  |  |  |
| Paradise | -0.074 | 0.406 |  |  |  |
| Rice | -0.435 | 0.477 |  |  |  |
| Slide | 0.875 | 0.405 |  |  |  |
| Tea | -0.799 | 0.505 |  |  |  |
| Thomas | -0.109 | 0.176 |  |  |  |
| Witch-Poomacha | 0.146 | 0.171 |  |  |  |
| Woolsey | 0.523 | 0.157 |  |  |  |

**S4 Table. Coefficient estimates and likelihood ratio test p-values for explanatory variables tested in the individual fire models using the Early Sales dataset.** Only fires with a significant effect of price are shown. The *r^2^*s for the Cedar, Grand Prix-Old, and Grass Valley fires are 0.13, 0.10, and 0.56, respectively. TPI refers to the topographic position index within a 500m window.

|  | **Cedar Fire 2003** | | | | | **Grand Prix-Old Fire 2003** | | | | | **Grass Valley Fire 2007** | | | | |
| --- | --- | --- | --- | --- | --- | --- | --- | --- | --- | --- | --- | --- | --- | --- | --- |
|  | **Coef.**  **Est.** | **Std. Error** | **LR Chisq** | **Df** | **Pr**  **(>Chisq)** | **Coef.**  **Est.** | **Std. Error** | **LR Chisq** | **Df** | **Pr (>Chisq)** | **Coef.**  **Est.** | **Std. Error** | **LR Chisq** | **Df** | **Pr**  **(>Chisq)** |
| Intercept | 4.472 | 1.575 |  |  |  | 5.385 | 3.107 |  |  |  | 109.448 | 1389.445 |  |  |  |
| ln(Price) | -0.710 | 0.125 | 31.554 | 1 | **<10^-6^** | -0.956 | 0.249 | 16.322 | 1 | **5.4x10^-5^** | -2.907 | 1.588 | 3.865 | 1 | **0.049** |
| Station Distance | 0.167 | 0.023 | 61.246 | 1 | <**10^-6^** | 0.033 | 0.035 | 0.870 | 1 | 0.351 | -0.025 | 0.876 | 0.001 | 1 | 0.977 |
| TPI | 0.014 | 0.003 | 17.252 | 1 | **<10^-6^** | -0.016 | 0.007 | 5.645 | 1 | **0.018** | 0.111 | 0.089 | 1.646 | 1 | 0.200 |
| Sales Year | -0.027 | 0.030 | 0.771 | 1 | 0.380 | -0.025 | 0.049 | 0.259 | 1 | 0.611 | 0.486 | 0.263 | 4.053 | 1 | **0.044** |
| Slope | 0.054 | 0.011 | 23.98 | 1 | **<10^-6^** | 0.070 | 0.019 | 13.8710 | 1 | **0.00020** | 0.194 | 0.146 | 2.021 | 1 | 0.155 |
| Elevation | 0.0004 | 0.0002 | 0.022 | 1 | 0.881 | 0.002 | 0.001 | 5.770 | 1 | **0.016** | -0.068 | 0.058 | 1.433 | 1 | 0.231 |
| Road Density | 323.200 | 67.320 | 23.023 | 1 | **1.6x10^-6^** | 577.900 | 116.500 | 32.91 | 1 | **<10^-6^** | 3447.076 | 3544.741 | 1.003 | 1 | 0.316 |
| Fire Hazard - High | -0.189 | 0.088 | 4.489 | 1 | **0.034** | -0.041 | 0.229 | 0.032 | 1 | 0.858 | -0.071 | 0.541 | 0.017 | 1 | 0.896 |
| Cover Type |  |  | 8.595 | 3 | **0.035** |  |  | 9.624 | 3 | **0.022** |  |  | 16.713 | 3 | **0.00081** |
| Barren | 0.063 | 0.204 |  |  |  | -0.741 | 0.372 |  |  |  | -8.955 | 3897.104 |  |  |  |
| Forest | 0.541 | 0.211 |  |  |  | -0.744 | 0.492 |  |  |  | -9.954 | 2004.529 |  |  |  |
| Herbaceous | -0.880 | 0.475 |  |  |  | 1.684 | 0.577 |  |  |  | 8.836 | 1385.841 |  |  |  |
| Shrub | 0.276 | 0.199 |  |  |  | -0.200 | 0.360 |  |  |  | 10.073 | 1385.841 |  |  |  |

**S5 Table. Coefficient estimates and likelihood ratio test p-values for variables in the Includes-Recession dataset.** Residual deviance was 5956 on 8690 degrees of freedom, with pseudo-r^2^ = 0.085.

|  | **Coefficient Estimate** | **Coefficient Std. Error** | **LR Chisq** | **Df** | **Pr(>Chisq)** |
| --- | --- | --- | --- | --- | --- |
| **Intercept** | 2.004 | 1.514 |  |  |  |
| ln(Price) | -0.314 | 0.112 | 8.519 | 1 | **0.0035** |
| **Fire Identity** |  |  | 77.049 | 9 | <**10^-6^** |
| Cedar | 2.637 | 1.977 |  |  |  |
| Grand Prix – Old | 10.898 | 2.783 |  |  |  |
| Grass Valley | -8.351 | 4.582 |  |  |  |
| Harris | 0.568 | 3.662 |  |  |  |
| Paradise | -4.399 | 5.242 |  |  |  |
| Slide | -4.359 | 4.611 |  |  |  |
| Tea | 20.344 | 9.504 |  |  |  |
| Thomas | -1.298 | 2.201 |  |  |  |
| Witch-Poomacha | -5.726 | 2.305 |  |  |  |
| Woolsey | -10.310 | 2.069 |  |  |  |
| **Station Distance (mins driving)** | 0.072 | 0.010 | 49.850 | 1 | <**10^-6^** |
| **Topographic Position Index (500m)** | 0.012 | 0.002 | 49.621 | 1 | <**10^-6^** |
| Sales Year | -0.021 | 0.019 | 1.284 | 1 | 0.257 |
| **Slope in 500m moving window** | 0.038 | 0.005 | 48.061 | 1 | <**10^-6^** |
| Elevation | 0.0003 | 0.0002 | 3.208 | 1 | 0.073 |
| Road Density (km/km2) | 9.637 | 25.965 | 0.138 | 1 | 0.711 |
| **Fire Hazard – High** | -0.145 | 0.049 | 8.464 | 1 | **0.0036** |
| **Cover Type** |  |  | 23.055 | 3 | **3.93x10^-5^** |
| Urban, Ag, Barren, Water | -0.201 | 0.091 |  |  |  |
| Forest | 0.063 | 0.115 |  |  |  |
| Herbaceous | -0.195 | 0.164 |  |  |  |
| Shrub | 0.333 | 0.088 |  |  |  |
| **ln(Price) x (Fire Identity)** |  |  | 76.075 | 9 | <**10^-6^** |
| Cedar | -0.238 | 0.150 |  |  |  |
| Grand Prix – Old | -0.882 | 0.221 |  |  |  |
| Grass Valley | 0.670 | 0.351 |  |  |  |
| Harris | -0.072 | 0.281 |  |  |  |
| Paradise | 0.258 | 0.408 |  |  |  |
| Slide | 0.312 | 0.368 |  |  |  |
| Tea | -1.298 | 0.676 |  |  |  |
| Thomas | 0.152 | 0.162 |  |  |  |
| Witch-Poomacha | 0.376 | 0.170 |  |  |  |
| Woolsey | 0.724 | 0.149 |  |  |  |

**S6 Table.** **Coefficient estimates and likelihood ratio test p-values for explanatory variables in the individual fire models using the Includes-Recession dataset.** Only fires with a significant price effect are shown. The *r^2^*s for the Cedar, Grand Prix-Old, and Tea fires are 0.14, 0.10, and 0.44, respectively. TPI refers to the topographic position index within a 500m window.

|  | **Cedar Fire 2003** | | | | | **Grand Prix-Old Fire 2003** | | | | | **Tea Fire 2008** | | | | |
| --- | --- | --- | --- | --- | --- | --- | --- | --- | --- | --- | --- | --- | --- | --- | --- |
|  | **Coef.**  **Est.** | **Std. Error** | **LR Chisq** | **Df** | **Pr**  **(>Chisq)** | **Coef.**  **Est.** | **Std. Error** | **LR Chisq** | **Df** | **Pr (>Chisq)** | **Coef.**  **Est.** | **Std. Error** | **LR Chisq** | **Df** | **Pr**  **(>Chisq)** |
| Intercept | 3.953 | 1.618 |  |  |  | 4.766 | 3.270 |  |  |  | -2.524 | 26.698 |  |  |  |
| ln(Price) | -0.684 | 0.129 | 27.68 | 1 | **<10^-6^** | -0.980 | 0.263 | 15.22 | 1 | **9.6x10^-5^** | -3.199 | 1.458 | 9.599 | 1 | **0.0019** |
| Station Distance | 0.193 | 0.024 | 72.47 | 1 | **<10^-6^** | 0.038 | 0.037 | 0.971 | 1 | 0.324 | 1.452 | 1.179 | 1.871 | 1 | 0.171 |
| TPI | 0.013 | 0.003 | 13.78 | 1 | **0.00021** | -0.017 | 0.008 | 4.766 | 1 | **0.029** | -0.089 | 0.066 | 2.393 | 1 | 0.122 |
| Sales Year | -0.020 | 0.034 | 0.349 | 1 | 0.555 | 0.013 | 0.055 | 0.058 | 1 | 0.810 | -0.565 | 0.432 | 2.073 | 1 | 0.150 |
| Slope | 0.0512 | 0.011 | 19.62 | 1 | **9.5x10^-6^** | 0.071 | 0.020 | 12.95 | 1 | **0.00032** | -0.033 | 0.236 | 0.020 | 1 | 0.888 |
| Elevation | 0.00005 | 0.0002 | 0.038 | 1 | 0.845 | 0.002 | 0.001 | 5.514 |  | **0.019** | 0.070 | 0.046 | 3.470 | 1 | 0.062 |
| Road Density | 321.300 | 69.810 | 21.123 | 1 | **4.3x10^-6^** | 644.100 | 130.400 | 34.293 | 1 | **<10^-6^** | 5984.312 | 4096.0 | 3.055 | 1 | 0.081 |
| Fire Hazard - High | -0.178 | 0.091 | 3.787 | 1 | 0.052 | -0.103 | 0.243 | 0.176 | 1 | 0.675 | -0.090 | 0.913 | 0.0098 | 1 | 0.921 |
| Cover Type |  |  | 9.206 | 3 | **0.027** |  |  | 6.757 | 3 | 0.080 |  |  | 0.579 | 1 | 0.447 |
| Barren | 0.056 | 0.208 |  |  |  | -0.693 | 0.411 |  |  |  | 0.790 | 1.075 |  |  |  |
| Forest | 0.576 | 0.213 |  |  |  | -0.664 | 0.523 |  |  |  | ^1^ | ^1^ |  |  |  |
| Herbaceous | -0.896 | 0.479 |  |  |  | 1.552 | 0.632 |  |  |  | ^1^ | ^1^ |  |  |  |
| Shrub | 0.264 | 0.202 |  |  |  | -0.196 | 0.387 |  |  |  | -0.790 | 1.075 |  |  |  |

^1^ Only barren and shrub cover types were present within the Tea Fire perimeter.

**S7 Table. Coefficient estimates and likelihood ratio test p-values for explanatory variables in the individual fire models for the Two-Year-Window dataset.** Only fires with a significant price effect are shown. The *r^2^*s for the Cedar and Grand Prix-Old are 0.22 and 0.16, respectively. TPI refers to the topographic position index within a 500m window.

|  | **Cedar Fire 2003** | | | | | **Grand Prix-Old Fire 2003** | | | | |
| --- | --- | --- | --- | --- | --- | --- | --- | --- | --- | --- |
|  | **Coef.**  **Est.** | **Std. Error** | **LR Chisq** | **Df** | **Pr**  **(>Chisq)** | **Coef.**  **Est.** | **Std. Error** | **LR Chisq** | **Df** | **Pr (>Chisq)** |
| Intercept | 7.406 | 6.067 |  |  |  | 15.600 | 321.500 |  |  |  |
| ln(Price) | -1.664 | 0.363 | 22.1133 | 1 | **2.6x10^-6^** | -2.053 | 0.711 | 11.0602 | 1 | **0.00088** |
| Station Distance | 0.295 | 0.073 | 19.8762 | 1 | **8.3x10^-6^** | 0.176 | 0.085 | 3.834 | 1 | 0.050 |
| TPI | 0.010 | 0.010 | 1.0443 | 1 | 0.307 | 0.012 | 0.023 | 0.278 | 1 | 0.598 |
| Sales Year | 0.782 | 0.393 | 5.692 | 1 | **0.017** | 0.071 | 0.355 | 0.041 | 1 | 0.839 |
| Slope | 0.033 | 0.035 | 0.867 | 1 | 0.352 | 0.123 | 0.067 | 3.468 | 1 | 0.063 |
| Elevation | -0.001 | 0.001 | 3.098 | 1 | 0.078 | 0.00003 | 0.002 | 0.00033 | 1 | 0.987 |
| Road Density | 647.900 | 214.300 | 9.2443 | 1 | **0.0024** | 151.100 | 260.800 | 0.357 | 1 | 0.550 |
| Fire Hazard - High | -0.412 | 0.241 | 2.8494 | 1 | 0.091 | 0.420 | 0.853 | 0.271 | 1 | 0.603 |
| Cover Type |  |  | 1.270 | 3 | 0.736 |  |  | 5.592 | 3 | 0.133 |
| Barren | -0.094 | 0.499 |  |  |  | 4.123 | 321.400 |  |  |  |
| Forest | 0.279 | 0.516 |  |  |  | -12.790 | 964.100 |  |  |  |
| Herbaceous | 0.263 | 1.010 |  |  |  | 5.857 | 321.400 |  |  |  |
| Shrub | -0.448 | 0.492 |  |  |  | 2.815 | 321.4 |  |  |  |

**S8 Table. Coefficient estimates and likelihood ratio test p-values for variables in the Assessor dataset.** Residual deviance was 11078 on 15307 degrees of freedom, with pseudo-r^2^ = 0.094.

|  | **Coefficient Estimate** | **Coefficient Std. Error** | **LR Chisq** | **Df** | **Pr(>Chisq)** |
| --- | --- | --- | --- | --- | --- |
| **Intercept** | 1.655 | 0.833 |  |  |  |
| **ln(Assessed Value)** | -0.212 | 0.062 | 11.406 | 1 | **0.00073** |
| **Fire Identity** |  |  | 38.773 | 12 | **0.00011** |
| Cedar | 2.432 | 1.564 |  |  |  |
| Corral | -8.752 | 3.563 |  |  |  |
| Creek | -3.422 | 2.512 |  |  |  |
| Grand Prix – Old | 5.163 | 2.793 |  |  |  |
| Harris | -0.859 | 2.882 |  |  |  |
| Jesusita | -0.751 | 3.139 |  |  |  |
| Lilac | -2.970 | 3.353 |  |  |  |
| Paradise | 1.797 | 4.873 |  |  |  |
| Rice | 12.000 | 3.818 |  |  |  |
| Tea | 2.644 | 1.805 |  |  |  |
| Thomas | -1.010 | 1.088 |  |  |  |
| Witch-Poomacha | -4.496 | 1.475 |  |  |  |
| Woolsey | -1.773 | 1.000 |  |  |  |
| **Station Distance (mins driving)** | 0.013 | 0.006 | 4.822 | 1 | **0.028** |
| **Topographic Position Index (500m)** | 0.016 | 0.001 | 187.84 | 1 | **<10^-6^** |
| **Recording Date** | -0.021 | 0.006 | 11.124 | 1 | **0.00085** |
| **Slope in 500m moving window** | 0.039 | 0.004 | 109.367 | 1 | **<10^-6^** |
| **Elevation** | -0.001 | 0.0002 | 40.764 | 1 | **<10^-6^** |
| **Road Density (km/km2)** | -196.400 | 17.130 | 134.804 | 1 | **<10^-6^** |
| **Fire Hazard – High** | -0.148 | 0.037 | 15.613 | 1 | **7.8x10^-5^** |
| **Cover Type** |  |  | 18.179 | 3 | **0.00040** |
| Urban, Ag, Barren, Water | 0.017 | 0.067 |  |  |  |
| Forest | 0.253 | 0.089 |  |  |  |
| Herbaceous | -0.443 | 0.121 |  |  |  |
| Shrub | 0.173 | 0.071 |  |  |  |
| **ln(Assessed Value) x (Fire Identity)** |  |  | 33.767 | 12 | **0.00073** |
| Cedar | -0.188 | 0.119 |  |  |  |
| Corral | 0.611 | 0.256 |  |  |  |
| Creek | 0.267 | 0.193 |  |  |  |
| Grand Prix – Old | -0.343 | 0.223 |  |  |  |
| Harris | 0.034 | 0.225 |  |  |  |
| Jesusita | 0.030 | 0.226 |  |  |  |
| Lilac | 0.238 | 0.254 |  |  |  |
| Paradise | -0.182 | 0.376 |  |  |  |
| Rice | -0.998 | 0.295 |  |  |  |
| Tea | -0.036 | 0.132 |  |  |  |
| Thomas | 0.136 | 0.082 |  |  |  |
| Witch-Poomacha | 0.305 | 0.110 |  |  |  |
| Woolsey | 0.125 | 0.075 |  |  |  |

**S9 Table. Coefficient estimates and likelihood ratio test p-values for explanatory variables in individual fire models using the Assessed-Value data.** Only the fires with significant coefficients for property value are shown. The *r^2^*s for the Cedar, Grand Prix-Old, Witch-Poomacha, Thomas, and Woolsey Fires are 0.10, 0.11, 0.16, 0.14, and 0.19, respectively. TPI refers to the topographic position index within a 500m window.

|  | **Cedar Fire 2003** | | | | | **Rice Fire 2007** | | | | | **Corral Fire 2007** | | | | |
| --- | --- | --- | --- | --- | --- | --- | --- | --- | --- | --- | --- | --- | --- | --- | --- |
|  | **Coef.**  **Est.** | **Std. Error** | **LR Chisq** | **Df** | **Pr**  **(>Chisq)** | **Coef.**  **Est.** | **Std. Error** | **LR Chisq** | **Df** | **Pr (>Chisq)** | **Coef.**  **Est.** | **Std. Error** | **LR Chisq** | **Df** | **Pr**  **(>Chisq)** |
| Intercept | 0.865 | 1.599 |  |  |  | 8.971 | 5.122 |  |  |  | -9.734 | 6.425 |  |  |  |
| ln(Assessed Value) | -0.332 | 0.119 | 7.725 | 1 | **0.0054** | -1.162 | 0.336 | 11.5271 | 1 | **0.00069** | 0.665 | 0.323 | 4.6094 | 1 | **0.032** |
| Station Distance | 0.097 | 0.021 | 25.1654 | 1 | **<10^-6^** | -0.085 | 0.096 | 0.8549 | 1 | 0.355 | -0.048 | 0.179 | 0.0717 | 1 | 0.789 |
| TPI | 0.014 | 0.004 | 13.5152 | 1 | **0.00024** | 0.001 | 0.015 | 0.0061 | 1 | 0.938 | -0.018 | 0.010 | 3.399 | 1 | 0.065 |
| Recording Date | -0.059 | 0.031 | 3.6779 | 1 | 0.055 | -0.086 | 0.069 | 1.5283 | 1 | 0.216 | -0.001 | 0.080 | 0.0002 | 1 | 0.990 |
| Slope | 0.064 | 0.012 | 27.9869 | 1 | **<10^-6^** | 0.020 | 0.050 | 0.162 | 1 | 0.687 | -0.045 | 0.052 | 0.748 | 1 | 0.387 |
| Elevation | 0.00003 | 0.0003 | 0.0146 | 1 | 0.904 | 0.006 | 0.004 | 2.5344 |  | 0.111 | 0.008 | 0.006 | 1.9153 | 1 | 0.166 |
| Road Density | 193.900 | 74.280 | 6.7823 | 1 | **0.0092** | 485.636 | 472.4 | 1.185 | 1 | 0.276 | -284.400 | 1676.000 | 0.0289 | 1 | 0.865 |
| Fire Hazard - High | -0.112 | 0.104 | 1.1541 | 1 | 0.283 | -0.168 | 0.340 | 0.2363 | 1 | 0.627 | -0.942 | 0.335 | 8.2919 | 1 | **0.0040** |
| Cover Type |  |  | 2.5645 | 3 | 0.464 |  |  | 3.2296 | 3 | 0.358 |  |  | 4.0581 | 3 | 0.131 |
| Barren | -0.247 | 0.189 |  |  |  | 0.168 | 0.513 |  |  |  | 0.321 | 0.527 |  |  |  |
| Forest | 0.140 | 0.203 |  |  |  | 1.172 | 0.639 |  |  |  | -1.188 | 0.645 |  |  |  |
| Herbaceous | 0.037 | 0.357 |  |  |  | -0.610 | 0.854 |  |  |  | ^1^ | ^1^ |  |  |  |
| Shrub | 0.071 | 0.182 |  |  |  | -0.730 | 0.913 |  |  |  | 0.867 | 0.611 |  |  |  |

^1^ Only barren, forest, and shrub cover types were present within the Corral Fire perimeter.

**S9 Table continued.**

|  | **Tea 2008** | | | | | **Woolsey Fire 2018** | | | | | | | | |  |
| --- | --- | --- | --- | --- | --- | --- | --- | --- | --- | --- | --- | --- | --- | --- | --- |
|  | **Coef.**  **Est.** | **Std. Error** | **LR Chisq** | **Df** | **Pr (>Chisq)** | **Coef.Est.** | **Std. Error** | | **LR Chisq** | | **Df** | | **Pr (>Chisq)** | |  |
| Intercept | 14.060 | 157.900 |  |  |  | 3.549 | | 0.732 | |  | |  | |  | |
| ln(Assessed Value) | -0.433 | 0.158 | 7.8921 | 1 | **0.0050** | -0.176 | | 0.047 | | 13.78 | | 1 | | **0.00020** | |
| Station Distance | -0.221 | 0.166 | 1.796 | 1 | 0.180 | -0.039 | | 0.013 | | 9.68 | | 1 | | **0.0019** | |
| TPI | 0.016 | 0.008 | 4.495 | 1 | **0.034** | 0.003 | | 0.002 | | 1.71 | | 1 | | 0.191 | |
| Recording Date | -0.009 | 0.041 | 0.053 | 1 | 0.818 | -0.025 | | 0.010 | | 6.39 | | 1 | | **0.011** | |
| Slope | 0.172 | 0.035 | 29.93 | 1 | **<10^-6^** | 0.033 | | 0.006 | | 26.00 | | 1 | | **<10^-6^** | |
| Elevation | -0.005 | 0.003 | 2.0704 | 1 | 0.150 | 0.002 | | 0.0004 | | 24.87 | | 1 | | **<10^-6^** | |
| Road Density | -864.60 | 407.200 | 4.711 | 1 | **0.030** | -882.500 | | 42.550 | | 521.26 | | 1 | | **<10^-6^** | |
| Fire Hazard - High | -0.090 | 0.194 | 0.217 | 1 | 0.642 | -0.212 | | 0.065 | | 10.29 | | 1 | | **0.0013** | |
| Cover Type |  |  | 11.581 | 3 | **0.0090** |  | |  | | 2.27 | | 3 | | 0.519 | |
| Forest | -4.200 | 157.900 |  |  |  | 0.143 | | 0.176 | |  | |  | |  | |
| Herbaceous | -3.034 | 157.900 |  |  |  | 0.248 | | 0.266 | |  | |  | |  | |
| Shrub | 11.510 | 473.700 |  |  |  | -0.318 | | 0.428 | |  | |  | |  | |
| Barren | -4.280 | 157.900 |  |  |  | -0.072 | | 0.200 | |  | |  | |  | |

Effect of Year Built

The relationship between property value and probability of burn damage likely occurs because less affluent home owners have fewer resources with which to protect their properties. Changes to building codes in 1991 and 2008 were designed to make all homes – regardless of value – more likely to survive a fire. As intended, the probability of burn damage has been shown to increase with the age of a structure, with a notable decrease in burn probabilities occurring after the 2008 changes to building codes (Baylis and Boomhower 2021).

For about 95% of our observations (similar to Nolte et al., In Press), the Zillow dataset includes information on the year the structure was built. Thus, we analyzed the combined influences of year built and property value on two datasets. The first dataset started with the sales data used to explore five fires in the main paper. The second dataset started with the 13-fire assessed value dataset. These two datasets represent two approaches to data inclusion criteria: the five-fire dataset having relatively restrictive criteria and the 13-fire assessed value dataset having relatively liberal criteria. Adding year built as a variable meant that some of the data in both of these datasets could not be used. For example, for the sales data, over 50% of the burned structures list the year built *after* the fire; almost 24% of the assessed value burned structures had year built after the fire. In other words, with the rebuilding of damaged structures, the Zillow dataset does not retain information on what year the destroyed structure was built. Some of the unburned structures also had values of year built after the fire or after a given sale. None of these observations could be used to explore the simultaneous influences of property price and the year the structure was built. After removing these points, we were left with the number of observations shown in S10 Table.

We analyzed the effect of year built in two ways for both the sales and assessed value data, namely, as a linear relationship and as a factor with three periods in which a structure could be built: before 1991, between 1991-2008, and after 2008. For the five-fire sales data, there was a significant negative relationship between the probability of burn damage and linear year built (*χ*^2^ = 45.26, *df* = 1, *p* < 10^-6^; S11 Table) and the probability of damage and year built as a factor (*χ*^2^ = 35.48, *df* = 1, *p* < 10^-6^; S12 Table). In both models, there was also a significant negative relationship between price and the probability of burn damage (*χ*^2^ = 15.16, *df* = 1, *p* = 9.9 x 10^-5^ in S11 Table; and *χ*^2^ = 20.11, *df* = 1, *p* = 7.3 x 10^-6^ in S12 Table). While there was a significant interaction between price and wildfire identity, no models performed on any of the individual fires (that included year built as an explanatory variable) had a significant relationship between price and probability of burn damage.

For the assessed value data, there was a significant negative relationship between the probability of burn damage and linear year built (*χ*^2^ = 121.95, *df* = 1, *p* < 10^-6^; S13 Table) and the probability of damage and year built as a factor (*χ*^2^ = 79.50, *df* = 2, *p* < 10^-6^; S14 Table). In both models, there was also a significant negative relationship between assessed value and the probability of burn damage (*χ*^2^ = 4.20, *df* = 1, *p* = 0.040 in S13 Table; and *χ*^2^ = 7.25 *df* = 1, *p* = 0.0071 in S14 Table). When fires were explored individually (S15 Table), only the Tea Fire had a significant negative relationship between burn damage and assessed value, when year built was linear or a factor (*χ*^2^ = 11.19, *df* = 1, *p* = 0.00082 and *χ*^2^ = 12.20, *df* = 1, *p* = 0.00048, respectively). The Woolsey Fire also had a marginally significant negative relationship (*χ*^2^ = 2.91, *df* = 1, *p* = 0.088) between burn damage and assessed value when year built was a factor, where year built was significant (*χ*^2^ = 43.26, *df* = 2, *p* < 10^-6^).

Given that the Cedar and Grand Prix-Old Fires had a significant relationship between probability of burn damage and price across all of the subsets presented in S4, S6, and S7 Tables, we expected that the significant relationship would be robust to the addition of year built as an explanatory variable. However, we think that removing rebuilt properties likely biased our analysis, a problem that affected these early fires more than later fires. If we assume that lower priced structures are more likely to be damaged in a fire, then we assume that lower priced structures would be more likely to be rebuilt. We removed 92% and 67% of burned-property sales observations and 92% and 61% of assessed value observations in the Cedar and Grand Prix-Old fires respectively, because these properties had year built values indicative of reconstruction.

Consistent with our assumption that removing reconstructed properties would remove the lowest valued properties, the datasets used to explore the effects of year built had higher property values, especially for burned structures. Specifically, the mean natural log-price for the burned structures was 13.22 (which corresponds to approximately $549,000) for the five-fire dataset in the main paper and 13.72 ($911,000) for the dataset used to look at year built. For the unburned structures, the values were 13.29 ($590,000) and 13.34 ($623,000) for the dataset used in the main paper and the dataset used to look at year built, respectively. The removal of rebuilt structures shifted the natural log of property price of burned structures by 0.50, an increase large enough that the mean natural log-price for burned structures became *higher* than unburned structures in the dataset used to look at year built. Comparing the dataset with and without year built, there was a much smaller (0.05) increase in the average log price of the unburned structures in the dataset that included year built. A similar situation occurs when comparing the 13-fire assessed value dataset and the 9-fire assessed value dataset that includes year built. The mean natural log-assessed value for the burned structures was 13.47 (which corresponds to approximately $708,000) for the 13-fire dataset and 13.59 ($798,000) for the nine-fire dataset used to look at year built. For the unburned structures, the values were 13.51 ($737,000) and 13.55 ($767,000) for the 13-fire and nine-fire datasets, respectively. Given that the coefficients for property price and assessed value are both negative and significant in S11-S15 Tables, the inclusion of other topographic and environmental variables in the models accounts for the fact that higher priced properties are more likely to be built in wildfire-prone areas.

**S10 Table. Summary of available data to analyze the impact of year built and property price simultaneously.**

|  | **Number of Structures in Sales Data** | | | **Number of Structures in**  **Assessed Value Data** | | |
| --- | --- | --- | --- | --- | --- | --- |
| **Fire Name** | **Unburned** | **Burned** | **Median Year Built** | **Unburned** | **Burned** | **Median Year Built** |
| Cedar | 2523 | 27 | 1989 | 1570 | 23 | 1987 |
| Grand Prix-Old | 547 | 43 | 1962 | 72 | 28 | 1961 |
| Witch-Poomacha | 606 | 6 | 1990 | 212 | 23 | 1987 |
| Corral | ^1^ | ^1^ | ^1^ | 112 | 18 | 1980 |
| Tea | ^1^ | ^1^ | ^1^ | 117 | 20 | 1981 |
| Thomas | 328 | 101 | 1973 | 1185 | 427 | 1973 |
| Creek | ^1^ | ^1^ | ^1^ | 300 | 33 | 1954 |
| Lilac | ^1^ | ^1^ | ^1^ | 222 | 31 | 1990 |
| Woolsey | 1607 | 172 | 1984 | 5522 | 638 | 1981 |

^1^ There were not enough points to analyze this fire in the specified analysis.

**S11 Table. Coefficient estimates and likelihood ratio test p-values for explanatory variables in the five-fire, sales price dataset used to explore the influence of a linear relationship of year built on probability of burn damage.** Residual deviance was 2077 on 5939 degrees of freedom, with pseudo-r^2^ = 0.22.

|  | **Coefficient Estimate** | **Coefficient Std. Error** | **LR Chisq** | **Df** | **Pr(>Chisq)** |  |
| --- | --- | --- | --- | --- | --- | --- |
| **Intercept** | 60.590 | 7.631 |  |  |  |  |
| **ln(Price)** | -0.954 | 0.267 | 15.155 | 1 | **9.91x10^-5^** |  |
| **Fire Identity** |  |  | 32.328 | 4 | **1.64x10^-6^** |  |
| Cedar | -6.530 | 5.306 |  |  |  |  |
| Grand Prix-Old | 5.022 | 5.077 |  |  |  |  |
| Witch-Poomacha | 29.330 | 11.990 |  |  |  |  |
| Thomas | -10.730 | 3.858 |  |  |  |  |
| Woolsey | -17.090 | 3.676 |  |  |  |  |
| **Year Built** | -0.026 | 0.004 | 45.263 | 1 | **<10^-6^** |  |
| **Station Distance (mins driving)** | 0.072 | 0.019 | 13.006 | 1 | **0.00031** |  |
| **Topographic Position Index (500m)** | 0.017 | 0.003 | 31.303 | 1 | **<10^-6^** |  |
| Sales Year | -0.027 | 0.035 | 0.58 | 1 | 0.446 |  |
| **Slope in 500m moving window** | 0.022 | 0.009 | 6.025 | 1 | **0.014** |  |
| **Elevation** | -0.002 | 0.0005 | 23.721 | 1 | **1.1x10^-6^** |  |
| **Road Density (km/km2)** | -86.510 | 39.990 | 4.693 | 1 | **0.030** |  |
| Fire Hazard – High | -0.112 | 0.098 | 1.273 | 1 | 0.259 |  |
| Cover Type |  |  | 7.697 | 3 | 0.053 |  |
| Urban, Ag, Barren, Water | -0.027 | 0.194 |  |  |  |  |
| Forest | 0.071 | 0.296 |  |  |  |  |
| Herbaceous | -0.576 | 0.388 |  |  |  |  |
| Shrub | 0.532 | 0.212 |  |  |  |  |
| **ln(Price) x (Fire Identity)** |  |  | 34.862 | 4 | **<10^-6^** |  |
| Cedar | 0.413 | 0.408 |  |  |  |  |
| Grand Prix-Old | -0.408 | 0.407 |  |  |  |  |
| Witch-Poomacha | -2.312 | 0.921 |  |  |  |  |
| Thomas | 0.961 | 0.293 |  |  |  |  |
| Woolsey | 1.346 | 0.278 |  |  |  |  |

**S12 Table. Coefficient estimates and likelihood ratio test p-values for explanatory variables in the five-fire, sales price dataset used to explore three time windows in which structures could have been built: before 1991, between 1992-2008, and after 2008.** These time windows were chosen based on building codes, implemented in 1991 and 2008, that were designed to mitigate wildfire damage to properties. Residual deviance was 2087 on 5938 degrees of freedom, with pseudo-r^2^ = 0.21.

|  | **Coefficient Estimate** | **Coefficient Std. Error** | **LR Chisq** | **Df** | **Pr(>Chisq)** |
| --- | --- | --- | --- | --- | --- |
| **Intercept** | 12.200 | 3.569 |  |  |  |
| **ln(Price)** | -1.141 | 0.275 | 20.112 | 1 | **7.3x10^-6^** |
| **Fire Identity** |  |  | 42.118 | 4 | **<10^-6^** |
| Cedar | -6.737 | 5.538 |  |  |  |
| Grand Prix-Old | 6.009 | 5.209 |  |  |  |
| Witch-Poomacha | 33.160 | 12.590 |  |  |  |
| Thomas | -12.500 | 3.988 |  |  |  |
| Woolsey | -19.330 | 3.819 |  |  |  |
| **Year Built** |  |  | 35.476 | 2 | <**10^-6^** |
| Before 1991 | 0.730 | 0.193 |  |  |  |
| Built in 1992-2008 | -0.338 | 0.210 |  |  |  |
| After 2008 | -0.393 | 0.348 |  |  |  |
| **Station Distance (mins driving)** | 0.071 | 0.019 | 13.292 | 1 | **0.00027** |
| **Topographic Position Index (500m)** | 0.017 | 0.003 | 32.113 | 1 | <**10^-6^** |
| Sales Year | -0.021 | 0.035 | 0.368 | 1 | 0.544 |
| **Slope in 500m moving window** | 0.020 | 0.009 | 5.044 | 1 | **0.025** |
| **Elevation** | -0.002 | 0.0005 | 29.368 | 1 | **<10^-6^** |
| **Road Density (km/km2)** | -99.230 | 39.700 | 6.270 | 1 | **0.012** |
| Fire Hazard – High | 0.151 | 0.097 | 2.363 | 1 | 0.124 |
| **Cover Type** |  |  | 5.719 | 3 | 0.126 |
| Urban, Ag, Barren, Water | -0.103 | 0.189 |  |  |  |
| Forest | 0.165 | 0.284 |  |  |  |
| Herbaceous | -0.464 | 0.379 |  |  |  |
| Shrub | 0.401 | 0.208 |  |  |  |
| **ln(Price) x (Fire Identity)** |  |  | 44.447 | 4 | <**10^-6^** |
| Cedar | -0.420 | 0.426 |  |  |  |
| Grand Prix-Old | -0.477 | 0.417 |  |  |  |
| Witch-Poomacha | -2.603 | 0.967 |  |  |  |
| Thomas | 1.101 | 0.303 |  |  |  |
| Woolsey | 1.560 | 0.289 |  |  |  |

**S13 Table. Coefficient estimates and likelihood ratio test p-values for variables in the Assessor dataset with year built as a linear term.** Residual deviance was 6657 on 12503 degrees of freedom, with pseudo-r^2^ = 0.178.

|  | **Coefficient Estimate** | **Coefficient Std. Error** | **LR Chisq** | **Df** | **Pr(>Chisq)** |
| --- | --- | --- | --- | --- | --- |
| **Intercept** | 46.640 | 4.106 |  |  |  |
| **ln(Assessed Value)** | -0.190 | 0.092 | 4.203 | 1 | **0.040** |
| **Fire Identity** |  |  | 20.762 | 8 | **0.0078** |
| Cedar | 0.756 | 4.316 |  |  |  |
| Corral | -4.510 | 4.385 |  |  |  |
| Creek | -4.297 | 2.886 |  |  |  |
| Grand Prix – Old | 3.171 | 4.160 |  |  |  |
| Lilac | -2.608 | 3.613 |  |  |  |
| Tea | 11.050 | 3.314 |  |  |  |
| Thomas | -1.108 | 1.448 |  |  |  |
| Witch-Poomacha | 0.382 | 3.433 |  |  |  |
| Woolsey | -2.830 | 1.332 |  |  |  |
| **Year Built** | -0.023 | 0.002 | 121.953 |  | **<10^-6^** |
| **Station Distance (mins driving)** | 0.042 | 0.011 | 14.315 | 1 | **0.00015** |
| **Topographic Position Index (500m)** | 0.020 | 0.002 | 167.187 | 1 | **<10^-6^** |
| **Sales Year** | -0.020 | 0.007 | 7.167 | 1 | **0.0074** |
| **Slope in 500m moving window** | 0.026 | 0.005 | 29.238 | 1 | **<10^-6^** |
| **Elevation** | -0.002 | 0.0003 | 34.756 | 1 | **<10^-6^** |
| **Road Density (km/km2)** | -233.400 | 22.260 | 111.509 | 1 | **<10^-6^** |
| **Fire Hazard – High** | 0.199 | 0.051 | 14.785 | 1 | **0.00012** |
| **Cover Type** |  |  | 28.726 | 3 | **2.6x10^-6^** |
| Urban, Ag, Barren, Water | 0.427 | 0.116 |  |  |  |
| Forest | -0.629 | 0.174 |  |  |  |
| Herbaceous | -0.941 | 0.263 |  |  |  |
| Shrub | 0.577 | 0.133 |  |  |  |
| **ln(Assessed Value) x (Fire Identity)** |  |  | 20.150 | 8 | **0.0098** |
| Cedar | -0.181 | 0.331 |  |  |  |
| Corral | 0.296 | 0.318 |  |  |  |
| Creek | 0.349 | 0.222 |  |  |  |
| Grand Prix – Old | -0.212 | 0.334 |  |  |  |
| Lilac | 0.234 | 0.274 |  |  |  |
| Tea | -0.751 | 0.249 |  |  |  |
| Thomas | 0.191 | 0.110 |  |  |  |
| Witch-Poomacha | -0.162 | 0.258 |  |  |  |
| Woolsey | 0.236 | 0.100 |  |  |  |

**S14 Table. Coefficient estimates and likelihood ratio test p-values for variables in the Assessor dataset with year built as a factor.** Residual deviance was 6700 on 12502 degrees of freedom, with pseudo-r^2^ = 0.172.

|  | **Coefficient Estimate** | **Coefficient Std. Error** | **LR Chisq** | **Df** | **Pr(>Chisq)** |
| --- | --- | --- | --- | --- | --- |
| **Intercept** | 1.356 | 1.266 |  |  |  |
| **ln(Assessed Value)** | -0.251 | 0.093 | 7.247 | 1 | **0.0071** |
| **Fire Identity** |  |  | 22.57 | 8 | **0.0040** |
| Cedar | 1.922 | 4.479 |  |  |  |
| Corral | -6.051 | 4.421 |  |  |  |
| Creek | -3.042 | 2.838 |  |  |  |
| Grand Prix – Old | 4.880 | 4.130 |  |  |  |
| Lilac | -4.055 | 3.616 |  |  |  |
| Tea | 10.890 | 3.333 |  |  |  |
| Thomas | -1.595 | 1.456 |  |  |  |
| Witch-Poomacha | 0.272 | 3.571 |  |  |  |
| Woolsey | -3.218 | 1.351 |  |  |  |
| **Year Built** |  |  | 79.503 | 2 | **<10^-6^** |
| Before 1991 | 0.693 | 0.252 |  |  |  |
| Built in 1992-2008 | -0.178 | 0.255 |  |  |  |
| After 2008 | -0.515 | 0.496 |  |  |  |
| **Station Distance (mins driving)** | 0.039 | 0.011 | 13.114 | 1 | **0.00029** |
| **Topographic Position Index (500m)** | 0.020 | 0.002 | 169.75 | 1 | **<10^-6^** |
| **Sales Year** | -0.021 | 0.007 | 7.809 | 1 | **0.0052** |
| **Slope in 500m moving window** | 0.025 | 0.005 | 29.397 | 1 | **<10^-6^** |
| **Elevation** | -0.002 | 0.0003 | 46.211 | 1 | **<10^-6^** |
| **Road Density (km/km2)** | -259.200 | 22.240 | 137.486 | 1 | **<10^-6^** |
| **Fire Hazard – High** | -0.209 | 0.051 | 16.435 | 1 | **5.0x10^-5^** |
| **Cover Type** |  |  | 21.356 | 3 | **8.9x10^-5^** |
| Urban, Ag, Barren, Water | 0.373 | 0.115 |  |  |  |
| Forest | -0.012 | 0.170 |  |  |  |
| Herbaceous | -0.843 | 0.260 |  |  |  |
| Shrub | 0.482 | 0.131 |  |  |  |
| **ln(Assessed Value) x (Fire Identity)** |  |  | 22.142 | 8 | **0.0047** |
| Cedar | -0.283 | 0.343 |  |  |  |
| Corral | 0.403 | 0.320 |  |  |  |
| Creek | 0.282 | 0.218 |  |  |  |
| Grand Prix – Old | -0.335 | 0.332 |  |  |  |
| Lilac | 0.329 | 0.274 |  |  |  |
| Tea | -0.731 | 0.251 |  |  |  |
| Thomas | 0.230 | 0.110 |  |  |  |
| Witch-Poomacha | -0.161 | 0.269 |  |  |  |
| Woolsey | 0.265 | 0.102 |  |  |  |

**S15 Table. Coefficient estimates and likelihood ratio test p-values for explanatory variables in individual fire models using the Assessed-Value data and including year built.** Only the 2008 Tea Fire is presented because it is the only fire with a significant coefficient for property value. The *r^2^*s are 0.429 and 0.477, when year built is linear and a factor, respectively. TPI refers to the topographic position index within a 500m window.

|  | **Coef.**  **Est.** | **Std. Error** | **LR Chisq** | **Df** | **Pr**  **(>Chisq)** | **Coef.**  **Est.** | **Std. Error** | **LR Chisq** | **Df** | **Pr (>Chisq)** |
| --- | --- | --- | --- | --- | --- | --- | --- | --- | --- | --- |
| Intercept | 99.950 | 366.400 |  |  |  | 24.690 | 600.000 |  |  |  |
| ln(Assessed Value) | -1.159 | 0.392 | 11.1982 | 1 | **0.00082** | -1.236 | 0.401 | 12.2089 | 1 | **0.00048** |
| Year Built | -0.040 | 0.022 | 3.5029 |  | 0.061 |  |  | 9.0515 |  | **0.00263** |
| Before 1991 |  |  |  |  |  | 1.526 | 0.596 |  |  |  |
| Built 1992-2008 |  |  |  |  |  | -1.526 | 0.596 |  |  |  |
| Station Distance | 0.796 | 0.449 | 3.3391 | 1 | 0.068 | 0.684 | 0.451 | 2.4766 | 1 | 0.116 |
| TPI | 0.050 | 0.022 | 6.9563 | 1 | **0.0084** | 0.041 | 0.021 | 4.6637 | 1 | **0.031** |
| Sales Year | -0.124 | 0.094 | 1.804 | 1 | 0.179 | -0.162 | 0.103 | 2.6436 | 1 | 0.104 |
| Slope | 0.117 | 0.092 | 1.7396 | 1 | 0.187 | 0.137 | 0.098 | 2.149 | 1 | 0.143 |
| Elevation | -0.011 | 0.009 | 1.8047 | 1 | 0.179 | -0.010 | 0.010 | 1.222 | 1 | 0.269 |
| Road Density | -784.6 | 1084 | 0.5632 | 1 | 0.453 | -1420 | 1153 | 1.739 | 1 | 0.187 |
| Fire Hazard - High | -0.243 | 0.585 | 0.161 | 1 | 0.688 | 0.451 | 0.617 | 0.471 | 1 | 0.493 |
| Cover Type |  |  | 3.4715 | 3 | 0.324 |  |  | 3.676 | 3 | 0.299 |
| Barren | -4.058 | 363.900 |  |  |  | -4.213 | 599.900 |  |  |  |
| Forest | -2.608 | 363.900 |  |  |  | -3.557 | 599.900 |  |  |  |
| Herbaceous | 11.400 | 1092 |  |  |  | 13.120 | 1800 |  |  |  |
| Shrub | -4.735 | 363.9 |  |  |  | -5.352 | 599.9 |  |  |  |


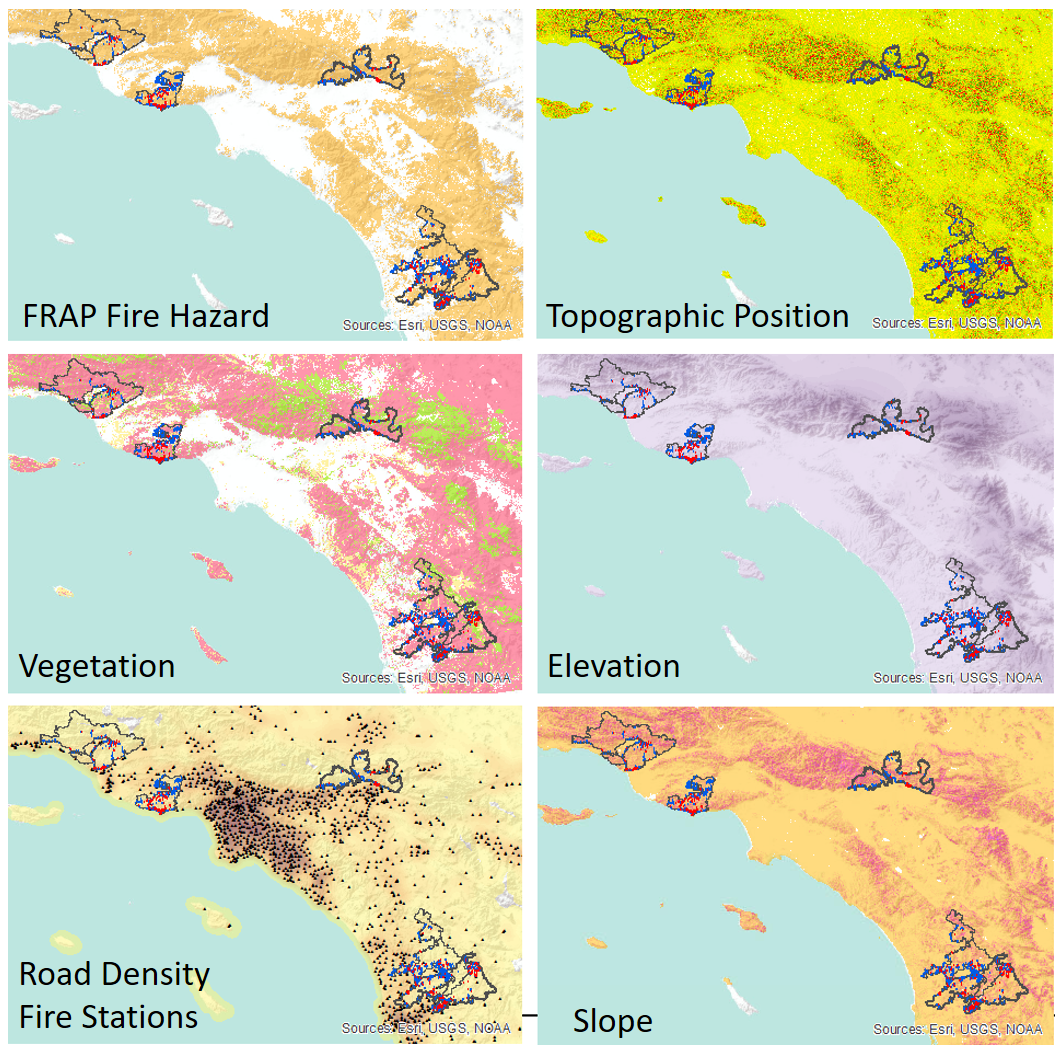


**S2 Fig. Maps of covariate values for each of the variables and including the fire perimeters and burned (red) and unburned (blue) points.** High Fire Hazard areas are shown in orange. Topographic position values shown vary from -100 (valleys) in green to 100 (peaks) in red. Vegetation categories are: red for shrublands, green for forest, yellow for herbaceous, and gray for barren. Higher elevations are shown in darker purple. Higher road density is shown in darker brown, with fire station locations shown in black dots. Steeper slopes are shown in red. Topographic base map provided by ESRI (2023).

**Supplemental Information References**

Alexandre Patricia M., Stewart Susan I., Keuler Nicholas S., Clayton Murray K., Mockrin Miranda H., Bar‐Massada Avi, Syphard Alexandra D., and Radeloff Volker C. 2016. Factors related to building loss due to wildfires in the conterminous United States. Ecological Applications 26:2323–2338.

Baylis, P., and J. Boomhower. 2021. Mandated vs. voluntary adaptation to natural disasters: the case of U.S. wildfires. National Bureau of Economic Research Working Paper:29621.

CALFIRE Incident Reports

2000:<https://web.archive.org/web/20020604173314/http://www.fire.ca.gov/FireEmergencyResponse/2000FireSeason/LF2001_041901.PDF>

2001:<https://web.archive.org/web/20040804050616/http://www.fire.ca.gov/FireEmergencyResponse/HistoricalStatistics/PDF/LF2001LISA.pdf>

2002:<https://web.archive.org/web/20171208215137/http://www.fire.ca.gov/fire_protection/downloads/LF2002.pdf>

2003:<https://web.archive.org/web/20181125181237/http://www.fire.ca.gov/fire_protection/downloads/LF2003final.pdf>

2004:<https://web.archive.org/web/20181125181214/http://www.fire.ca.gov/fire_protection/downloads/LARGEFIRE2004final.pdf>

2005:<https://web.archive.org/web/20160304193148/http://cdfdata.fire.ca.gov/pub/cdf/images/incidentstatsevents_113.pdf>

2006:<https://web.archive.org/web/20161118060633/http://cdfdata.fire.ca.gov/pub/cdf/images/incidentstatsevents_123.pdf>

2007:<https://web.archive.org/web/20160304200939/http://cdfdata.fire.ca.gov/pub/cdf/images/incidentstatsevents_167.pdf>

2008:<https://web.archive.org/web/20161118020210/http://cdfdata.fire.ca.gov/pub/cdf/images/incidentstatsevents_179.pdf>

2009:<https://web.archive.org/web/20160305023031/http://cdfdata.fire.ca.gov/pub/cdf/images/incidentstatsevents_178.pdf>

2010:<https://web.archive.org/web/20160304200453/http://cdfdata.fire.ca.gov/pub/cdf/images/incidentstatsevents_185.pdf>

2011:<https://web.archive.org/web/20160824154833/http://cdfdata.fire.ca.gov/pub/cdf/images/incidentstatsevents_214.pdf>

2012:<https://web.archive.org/web/20181125175809/http://cdfdata.fire.ca.gov/pub/cdf/images/incidentstatsevents_255.pdf>

2013:<https://web.archive.org/web/20150910201400/http://cdfdata.fire.ca.gov/pub/cdf/images/incidentstatsevents_250.pdf>

2014:<https://web.archive.org/web/20171208203602/http://cdfdata.fire.ca.gov/pub/cdf/images/incidentstatsevents_263.pdf>

2015:<https://web.archive.org/web/20171211213657/http://calfire.ca.gov/downloads/redbooks/2015_Redbook/2015_Redbook_300Acres-and-greater.pdf>

2016:<https://web.archive.org/web/20171211213701/http://cdfdata.fire.ca.gov/pub/cdf/images/incidentstatsevents_272.pdf>

2017-2019: <https://www.fire.ca.gov/incidents/2017>

Nolte, C., K. J. Boyle, A. Chaudhry, C. Clapp, D. Guignet, H. Hennighausen, I. Kushner, Y. Liao, S. Mamun, A. Pollack, J. Richardson, S. Sundquist, K. Swedberg, and J. H. Uhl. In Press. Data Practices for Studying the Impacts of Environmental Amenities and Hazards with Nationwide Property Data. Land Economics.

Syphard, A. D., H. Rugustian-Romsos, M. Mann, E. E. Conlisk, M. A. Moritz, and D. D. Ackerly. 2019. The relative influence of climate and housing development on current and projected future fire patterns and structure loss across three California landscapes. Global Environmental Change 56:41–55.

ZTRAX. 2018. Zillow Transaction and Assessor Dataset Frequently Asked Questions: [https://www.zillow.com/research/ztrax/ztrax-faqs/](https://www.zillow.com/research/ztrax/ztrax-faqs/%20)
